# Structure of human green cone opsin yields insights into mechanisms underlying the rapid decay of its active, signaling state

**DOI:** 10.1073/pnas.2516318122

**Published:** 2025-12-02

**Authors:** Weekie Yao, Jonathan F. Fay, David L. Farrens

**Affiliations:** ^a^Department of Chemical Physiology and Biochemistry, Oregon Health and Science University, Portland, OR 97239; ^b^Department of Biochemistry and Molecular Biology, University of Maryland Baltimore, Baltimore, MD 21201

**Keywords:** cone opsin structure, cone opsin function, GPCRs, visual signal transduction

## Abstract

Cone opsins enable daylight vision and color discrimination. Like their dim-light cousin rhodopsin (Rho) found in rod cells, they use a covalently attached retinal ligand to sense light and initiate visual phototransduction by activating G proteins. Unfortunately, we know less about their structural properties, in part because their activated state is unstable—cone opsins release their retinal agonist within seconds after light activation, ~100× faster than Rho. To determine what causes this rapid release and how it affects G protein activation, we solved the structure of active-state, wild-type human green cone opsin (GCO_WT_) stabilized with a mini-G protein and then compared its structural and biophysical properties to Rho. Our results reveal unique features in the active-state GCO_WT_ structure. These include i) a larger water channel connected to a larger retinal binding cavity, ii) a larger “hole” near the retinal Schiff base that could facilitate both retinal escape and water access; and iii) a potential anionic residue, E102, that lies within ~3.6 Å of the Schiff base. Our biophysical assays show that neutralizing E102 (mutant GCO_E102Q_) slows retinal release (~8×) from the receptor and increases G protein activation. Surprisingly, our kinetic studies suggest that entropic factors are the main cause for the faster retinal release from activated GCO_WT_. These unique attributes in GCO_WT_ likely facilitate its function in bright daylight. These results support the proposal that rapid retinal release from an active-state cone opsin helps prevent signal saturation and enables rapid resetting of the receptor.

## Significance

Our daylight vision uses cone opsins. Unfortunately, we know less about these light-sensitive GPCRs, in part because their active state is unstable and decays within seconds (~100× faster than rhodopsin). We studied wild-type human green cone opsin (GCO_WT_), probing both the cause and effect of this instability using structural and biochemical methods. We find that entropic differences cause the faster release of the agonist (retinal) from active-state GCO_WT_, a process aided by its more porous active structure, which features a highly conserved anionic residue near the retinal. Moreover, a GCO mutation that slows active state decay increases its signaling ability, suggesting these features enable the cone opsins to function under bright light without signal saturation.

## Visual Opsins All Share a Similar Signaling Mechanism—The Visual Signal Cascade.

Our ability to see depends on visual opsins, light-sensitive G protein-coupled receptors (GPCRs) that can detect a wide range of light intensities and colors. In bright light, we use three different cone opsins (1)—green cone opsin (GCO), red cone opsin (RCO), and blue cone opsin (BCO). At night (or other dim-light conditions), we use a different opsin, called rhodopsin (Rho) (2, 3). Interestingly, all visual opsins use retinylidene chromophores, but vary their amino acid sequences around this ligand to tune their response to different light (4).

In the dark, visual opsins are kept inactive by a bound retinal isomer 11-cis-retinal (11CR). They are “turned on” when light converts 11CR into an agonist, all-trans-retinal (ATR). The resulting active protein conformation, called meta II (MII), binds and activates a G protein, called transducin (Gt), thus starting the visual signaling cascade. Active MII is “turned off” (inactivated) when the protein arrestin binds, or when the link attaching ATR is hydrolyzed and ATR agonist is released. The latter enables the inactive apoprotein, opsin, to then bind a new 11CR and restart the visual cycle.

Understanding these processes in visual opsins is challenging. Unlike other GPCRs, visual opsins bind their ligand using both noncovalent interactions and a covalent (Schiff base) link between the retinal and a conserved Lys residue in the receptor. Thus, the properties of visual opsins depend on how these different interactions control retinal absorbance and how they affect retinal binding affinity.

## All Visual Opsins Activate G proteins, But Not to the Same Extent.

While all mammalian visual opsins use the signal cascade described above, cone opsins show a lower overall ability to activate G proteins (5, 6). This lower activity could be due to differences in how cone opsins interact with G proteins, although sequences in the signal coupling domain are conserved among visual opsins (7). Alternatively, this lower activity could be due to the instability of cone opsins in the MII state (6, 8–11)—once activated, they release the ATR agonist within seconds compared to tens of minutes for rhodopsin (3). To date, it has not been possible to assess these different possibilities, as the structure of a wild-type cone opsin has not been available, and biophysical assays to monitor cone opsins’ stability have been challenging.

## Goals and Overview of the Present Study.

Our goal here was to identify the underlying cause for faster active state decay and lower signaling of a cone opsin. To do this, we set out to directly compare the structural, functional, and biochemical properties of wild-type human green cone opsin (GCO_WT_) with wild-type human rhodopsin (Rho_WT_) and bovine rhodopsin (bRho_WT_).

First, we determined the structure of an active GCO_WT_/G protein complex, and compared it to published structures of active bRho_WT_/G protein complexes (12). We find that both receptors bind the G protein Gα C-terminal tail in a very similar way. However, our structure shows unique features in GCO_WT_ contribute to its rapid decay from the active, MII state. GCO_WT_ has a larger cavity around the retinal, a larger channel connecting to this cavity, a larger potential “exit hole” near the retinal Schiff base attachment site and poised directly below this, an anionic residue (E102), that is both highly conserved and unique to green and red cone opsins.

We then directly compared the functional properties of Rho_WT_ and GCO_WT_ and confirmed that active-state GCO_WT_ decays faster and activates fewer G proteins than Rho_WT_. Intriguingly, we find that neutralizing E102 (mutant GCO_E102Q_) results in slower active-state decay and increased G protein activation. Further analysis of the Schiff base hydrolysis mechanism suggests the faster hydrolysis rates in GCO are due to a lower entropic barrier, while the underlying energetics for hydrolysis are similar.

Together, our data show that GCO_WT_ has reduced signaling not because of *how* it interacts with G protein, but rather, *how long* it can maintain its active, signaling state. We propose that key features in GCO_WT_, like E102, enable the accelerated active-state decay of cone opsins. As has previously been proposed, the rapid decay of active cone opsins would enable them to rapidly reset and function under bright light conditions.

## Results

### Structure of ATR-Bound GCO_WT_ /G Protein Complex.

**Fig. 1.**
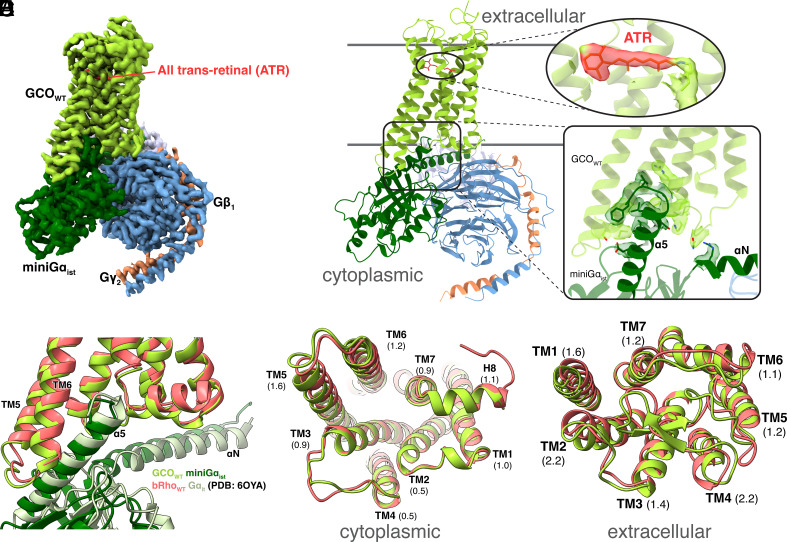
Cryo-EM structural analysis of active-state GCO_WT_ bound to G protein (miniGα_ist_-Gβ_1_-Gγ_2_) and its comparison to bovine rhodopsin (bRho_WT_). (*A*) Cryo-EM map of the GCO_WT_–miniGα_ist_ complex. Subunits are colored as follows: GCO_WT_ (green), all-trans-retinal (red), miniGα_ist_ (dark green), Gβ_1_ (blue), Gγ_2_ (orange), scFv16 (white). (*B*) Corresponding ribbon structure model of the GCO_WT_–miniGα_ist_ protein complex. Insets show expanded views of the retinal ligand (all-*trans-*retinal, ATR) and the main G protein interaction sites with cryo-EM densities shown as colored transparent surfaces. (*C*) Aligned ribbon structure (backbone) comparison of GCO_WT_ (green) bound to miniGα_ist_ (dark green) with bovine Rho_WT_ (bRho_WT_, PDB: 6OYA, red) bound to Gα_it_ (PDB:6OYA, light green). Rhodopsin-G protein structure (PDB: 6OYA) was aligned with GCO-G protein structure in ChimeraX. (*D* and *E*) Aligned ribbon structures comparing the backbone architecture of GCO_WT_ (green) and bRho_WT_ (red). Rmsd for each helix is shown Å in parentheses (*D*) View from the cytoplasmic (overall Cα-Cα rmsd: 1.16 Å) and (*E*) extracellular faces (overall Cα-Cα rmsd: 2.04 Å). Note: The bRho (receptor only from PDB: 6OYA) was aligned with GCO structure in ChimeraX, and the N terminus hidden for clarity.

We determined a cryogenic electron microscopy (cryo-EM) structure of GCO_WT_ bound to a Gi/Gt chimeric heterotrimeric signaling complex (Fig. 1) containing all-*trans-*retinal (ATR).

Our approach (*SI Appendix*, Figs. S1 and S2) used a well-established strategy (13–16) that involves coexpressing the GCO_WT_ with Gβ_1_, Gγ_2_, and a mini-Gα_i_ protein, then adding exogenous agonist (in this case, ATR) and purifying the resulting receptor/G protein complex. Excess ATR is added during protein expression and purification to stabilize the active-state GCO_WT_ /G protein complex. Note: The receptor and Gβ_1_ contained a C-terminal 1D4 epitope tag to both aid in 1D4 immunoaffinity purification and potentially facilitate stabilizing the complex during purification. Single chain antibody Fv16 has been shown to stabilize GPCR-G protein complex for structural studies (17) and is used here to further stabilize GCO_WT_-G protein complex. Our cryo-EM map of the GCO_WT_/G protein complex with covalently bound agonist ATR was obtained at global nominal resolution of 2.9Å (Fig. 1*A* and *SI Appendix*, Fig. S2 and Table S1).

Overall, the structure displays canonical features of active-state GPCR/G protein complex (Fig. 1). Among these are the outward movement of TM5 and TM6 that exposes a cytoplasmic pocket on the receptor (18, 19), into which the Gα C-terminus (α5 helix) binds and interacts with a “hydrophobic patch” on the receptor (20) (Fig. 1*B* and *SI Appendix*, Fig. S4). Fig. 1*C* and *SI Appendix*, Fig. S5 illustrate these same interactions are observed in the cryo-EM structure of wild-type bovine rhodopsin (referred to as bRho_WT_) bound to the G protein transducin [PDB:6OYA, (12)]. We chose this structure for our comparisons, because like our GCO_WT_ structure, it represents an unmodified wild-type receptor/G protein complex solved by cryo-EM to a similar resolution. A comparison of other opsin/G protein complexes, including a recent BioRXiv paper describing a GCO mutant (GCO_E129Q_) bound to Gi (21), are shown in *SI Appendix*, Fig. S5.

### Main Structural Differences between GCO_WT_ and bRho_WT_ Occur around the Retinal Binding Pocket.

Interestingly, the backbone structure of active-state GCO_WT_ is also very similar to active-state bRho_WT_ when viewed from the cytoplasmic face (Fig. 1*D*, Cα- Cα total rmsd: 1.16 Å). However, from an extracellular view, larger differences can be seen in the location of some loops and helices (Fig. 1*E*, Cα- Cα total rmsd: 2.04 Å; rmsd for individual helices shown in parentheses).

**Fig. 2.**
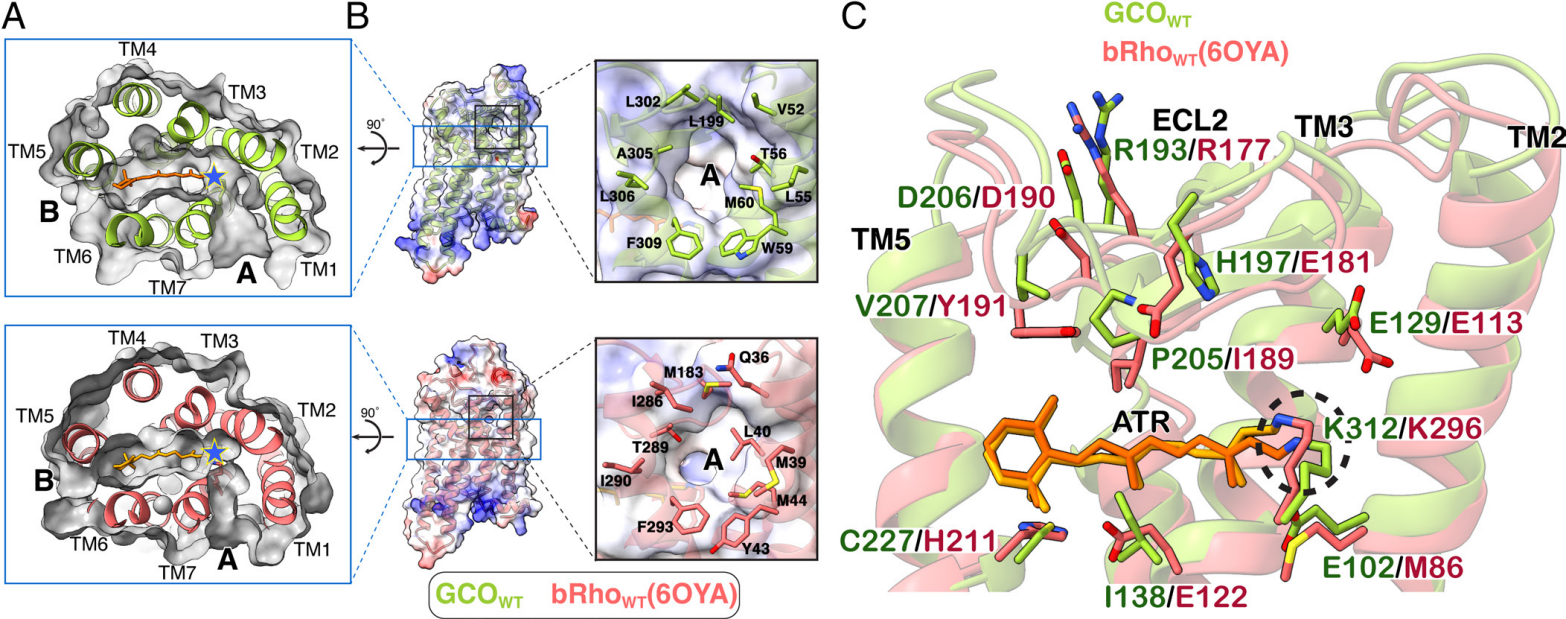
Structural comparisons of active-state human GCO_WT_ and bRho_WT_ (PDB:6OYA) reveal both similarities and differences around the retinal binding pocket. (*A*) Top–down view of both active-state structures (top = GCO_WT_, bottom = bRho_WT_) reveals that both retinal binding cavities are flanked by openings on two sides, referred to as Hole A and Hole B. Note that active-state GCO_WT_ has a larger retinal cavity and larger Hole A near the Schiff base (blue star). The images represent a horizontal slice (and 90° rotation) through the area of the receptor indicated by the blue box in (*B*). (*B*, *Left*) Side view of both active-state structures. (*Right*) Close-up of the Hole A showing the larger Hole A in active-state GCO_WT_ (*Top*) than in bRho_WT_ (*Bottom*). The Hole A images correspond to the black box on the full receptor (*Left*), with surrounding residues indicated and the surface colored by electrostatic surface potential calculated in ChimeraX (23). (*C*) Side-view comparing the retinal binding pocket and ECL2 of active-state GCO_WT_ (green) and bRho_WT_ (red). (Note: For clarity, TM1, TM6, and TM7 are not shown). Schiff bases are indicated (dashed black circle). Key similarities and differences between the receptors are discussed below in a clock-wise fashion, starting at the top of the figure. Similarities include the overall structure for the ECL2 loop, the location of a primary counterion (Glu) on TM3, and the retinal Schiff base linkage to Lys on TM7. Differences include alternate residues at specific sites. These similarities and differences are described in more detail in the Main Text. Of special interest is a unique Glu residue (E102^2.53^) on TM2 of GCO_WT_ that lies near to and below the retinal Schiff base.

A more detailed comparison of this region shows several noteworthy similarities and differences. Both receptors have two “holes” in the sides of the receptor (Fig. 2*A*). These openings, first discovered in active-state bRho_WT_ (22), have been denoted as “Hole A” (flanked by TM1 and TM7) and “Hole B” (flanked by TM5 and TM6). Interestingly, Hole A, which lies next to the retinal Schiff base, is significantly larger in GCO_WT_ (Fig. 2 *A* and *B*).

Other similarities inside the retinal binding pocket are shown in Fig. 2*C* and are discussed below in a roughly counterclockwise order. Note the similar structures for EL2, the loop connecting TM4 and TM5, which lies directly above the retinal. In both receptors, EL2 appears held together at the ends by an ion-pair (R193/D206 in GCO; R177/D190 in bRho_WT_) and is linked to TM3 by a conserved disulfide (C126^3.25^ - C203^45.50^ in GCO_WT_; C110^3.25^ - C187^45.50^ in bRho_WT_). [When possible, residue numbers include Ballesteros-Weinstein numbering in superscript to aid in comparison (24)].

Both receptors also share highly conserved residues that are located in the same positions. These include a Lys residue on TM7 that links to the retinal by a Schiff base (K312^7.42^ in GCO_WT_; K296^7.42^ in bRho_WT_). Interestingly, the Lys-retinal connection in GCO_WT_ has a more compact (“bent”) conformation due to the Schiff base being drawn toward E102^2.53^ (discussed further below). Across from the retinal attachment site, both receptors have a Glu residue on TM3 (E129^3.28^ in GCO_WT_; E113^3.28^ in bRho_WT_). In Rho, this Glu acts as the “primary counterion,” which stabilizes a protonated retinal Schiff base in the dark state receptor and enables long-wavelength absorbance.

Significantly, there are several key residues that while differing in identity, are located at the same position in both structures. In EL2, GCO_WT_ has a Pro (P205^45.52^) where bRho_WT_ has an Ile (I189^45.52^), and GCO_WT_ has a His (H197) where bRho_WT_ has a Glu (E181). This is noteworthy because E181 is highly conserved in rhodopsins, and is involved in rhodopsin photoactivation (25–27), whereas H197 is highly conserved in green and red cone opsins; and has been proposed to be part of a chloride (Cl−) binding site for these opsins in the dark-state (28). We do not see evidence for a Cl− (density) near H197, but this could be because Cl− binds with relatively low affinity (~0.6 mM) (28), or because it moved to a different location in the active-state structure. Regardless, it is interesting that H197 in GCO_WT_, like E181 in bRho_WT_, is pointed toward the retinal (Fig. 2*C*).

In TM3, GCO_WT_ has an Ile (I138^3.37^) instead of a conserved Glu found in bRho_WT_ (E122^3.37^), and in TM5, a Cys (C227^5.47^) in place of a His (H211^5.47^). This indicates that GCO_WT_ lacks the ability to form a hydrogen-bond equivalent to that thought to form between E122^3.37^ and H211^5.47^in active-state bRho_WT_ (29, 30).

**Fig. 3.**
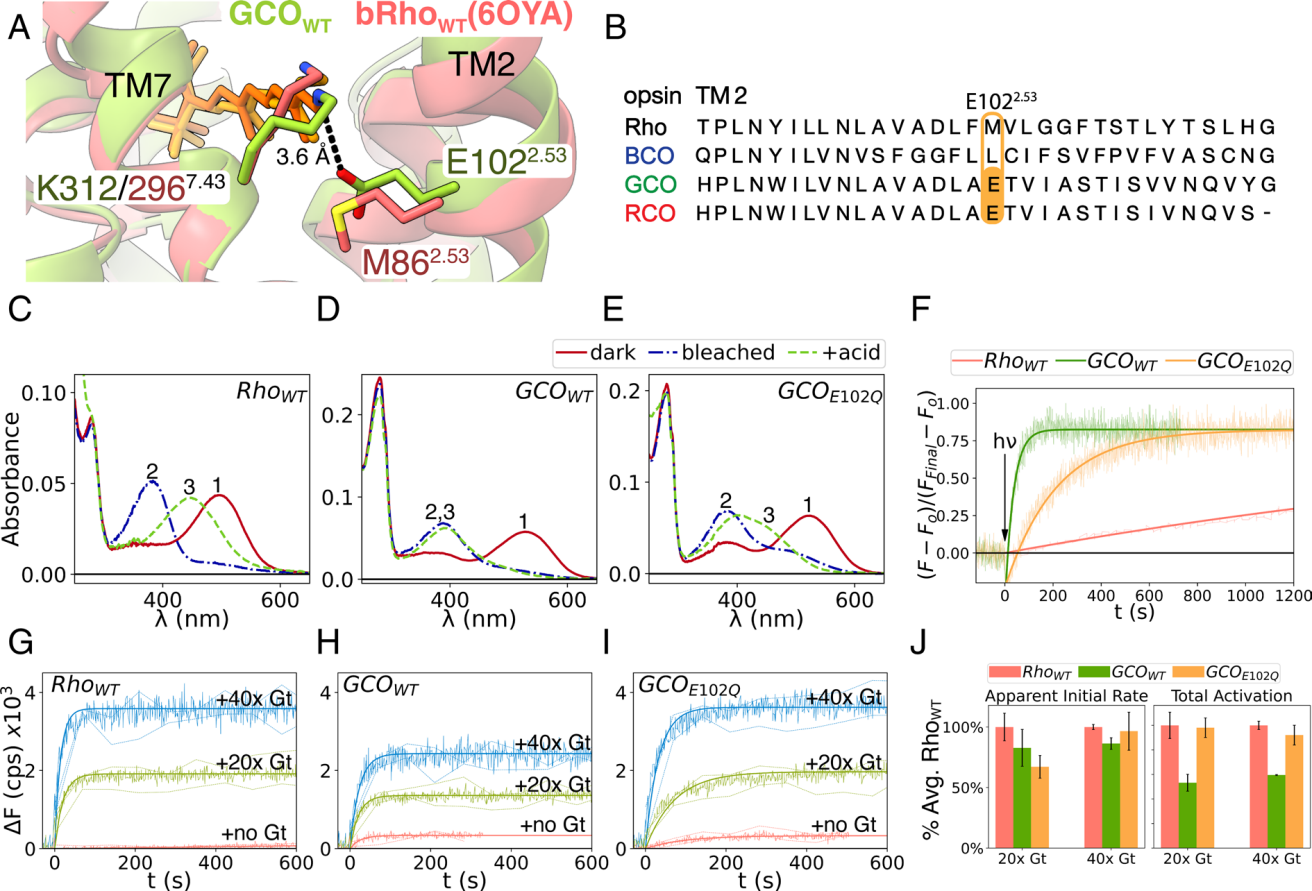
Active-state GCO_WT_ decays faster and has a reduced ability to activate G proteins than Rho_WT_, but neutralizing residue E102 (GCO_E102Q_) increases its active-state stability and total G protein activation. (*A*) Location of E102^2.53^ in active-state GCO_WT_ compared to M86^2.53^ in active-state bovine Rho_WT_ (bRho_WT_, PDB:6OYA). (*B*) Sequence alignment of TM2 in human visual opsins showing that residue E102^2.53^ only occurs in human green cone (GCO) and red cone opsins (RCO), but not in blue cone opsin (BCO) or Rho. (*C*–*E*) UV-vis absorbance spectra of purified human Rho_WT_ (*C*) GCO_WT_ (*D*) and GCO_E102Q_ (*E*). Spectra were measured in the dark 1) and then after light activation 2). Samples were also acidified and measured 3) to assess whether the retinal-Schiff base linkage is present, indicated by a 440 nm absorbing species. (*F*) Retinal release/MII decay measurements for Rho_WT_ (red), GCO_WT_ (green), and GCO_E102Q_ (yellow) at 10 °C. The assay detects the increase in tryptophan (Trp) fluorescence (330 nm) upon receptor light activation (hv) that accompanies the subsequent release of retinal from the receptor binding pocket. (*G*–*I*) G protein (Gt) activation assays of purified human Rho_WT_, GCO_WT_, and GCO_E102Q_. For each assay, the receptor was mixed with Gt, then irradiated for 2 s (t = 0), while the Trp fluorescence was monitored to measure the increase in Trp fluorescence from activated Gt when it exchanges GDP for GTPγS. The control (red line) shows Trp fluorescence increase due to retinal release from receptor alone (as in *F*), measured in the absence of Gt and GTPγS). Note: The higher levels of Trp fluorescence for the GCO_WT_ and GCO_E102Q_ controls samples are seen because they decay ~100× faster than Rho_WT_, and thus fully release their retinal during the span of the measurement (10 min). Measurements were carried out at 9 °C using 15 nM of the indicated opsin, and either 300 nM Gt and 5 µM GTPγS (20× Gt, green line) or 600 nM Gt and 10 µM GTPγS (40× Gt, blue line). The solid lines represent an average of multiple independent measurements (n = 3 to 5), with one SD of the average shown as the dotted lines. The fluorescence before light activation was averaged to obtain dark state levels, and then subtracted to obtain ΔF. (*J*) Initial rate and total activation estimated from results shown in (*G*–*I*). Apparent initial rates were estimated from linear regression of the initial fluorescence increase (30 s after hv). The total activation values were obtained by fitting the fluorescence increase to monoexponential rise to maxima.

However, perhaps the most intriguing difference lies in TM2, where GCO_WT_ has a Glu residue, (E102^2.53^) instead of the Met (M86^2.53^) seen in bRho_WT_. As shown in Fig. 3*A*, E102^2.53^ is poised very close to the retinal Schiff base (~3.6 Å) and is highly conserved and unique to green and red cone opsins (7, 31) (Fig. 3*B* and *SI Appendix*, Fig. S6). We tested the potential role of this residue in active-state stability and function as discussed below.

### Biochemical Studies Identify Factors That Impact Activated GCO Stability and Function.

We next assessed how the different active-state stabilities of human GCO_WT_, human GCO mutant GCO_E102Q_ and human Rho_WT_ could affect their function. For these studies, the receptors were expressed in COS-1 cells in the presence of β-ionone (instead of ATR) to help stabilize the receptor (32), then regenerated with 11CR, solubilized, and purified as described in the *Materials and Methods* and *SI Appendix*. We used identical conditions to prepare these samples, so that we could directly compare their spectral properties and their ability to activate G protein purified from retinas (transducin, or Gt). The results are described below.

#### Spectral analysis of the purified visual opsins before and after light activation.

In its purified dark-state, Rho_WT_ (Fig. 3*C*) absorbs with a λ_max_ at 500 nm (A_500nm_), as expected (33). Photoactivation with λ > 490 nm light yields an active MII form with a λ_max_ = 380 nm. Acidifying this MII species (A_380 nm_) results in almost quantitative conversion to A_440 nm_ species, which corresponds to a protonated retinal Schiff base, thus indicating the retinal linkage is still stably attached to the protein.

The purified dark-state GCO_WT_ (Fig. 3*D*) absorbs with a λ_max_ at 530 nm as previously reported (28), and contains at ~80% properly folded receptor, based on the protein to chromophore absorbance ratio (A_280_/A_530_) of ~3.3, vs. a theoretical maximal value of 2.5 (*Materials and Methods* for more details). Activating light (λ > 490 nm) converts the GCO_WT_ to a 380 nm absorbing MII species. In contrast to Rho_WT_, the GCO_WT_ MII species appears to rapidly decay, as subsequent acidification detects no protonated retinal Schiff base linkage (A_440 nm_ species), consistent with a fast decay of active-state GCO (34).

As noted in Fig. 2*C*, GCO_WT_ has a Glu residue (E129^3.28^) on TM3 where the primary counterion in bRho_WT_ is located (E113^3.28^). To confirm that E129^3.28^ is the primary counterion in GCO_WT_, we “neutralized” it, by creating and analyzing a mutant GCO_E129Q_. Our results (*SI Appendix*, Fig. S7*A*) show that dark-state GCO_E129Q_ binds 11CR but does not exhibit a long-wavelength “opsin” shift to 530 nm. Instead, it absorbs with a λ_max_ of ~380 nm, which upon acidification yields a ~440 nm absorbing protonated retinal Schiff base species, confirming 11CR binding to the receptor. This same behavior is seen when the primary counterion is neutralized in other opsins (35).

We also tested the effect of neutralizing residue E102^2.53^ on TM2. In contrast to GCO_E129Q_ above, purified dark-state GCO_E102Q_ exhibits a λ_max_ of ~520 nm (Fig. 3*E* and *SI Appendix*, Fig. S7*B*). This relatively small (~10 nm) blue-shift compared to GCO_WT_ shows that in the dark-state, E102 is close enough to slightly modulate 11CR absorbance (perhaps due to a difference in the overall polarity in that part of the binding pocket) but does not act as a primary counterion that can modulate the pKa of the retinal Schiff-base, since the E102Q mutation did not cause the type of large absorbance blue-shift that would be expected (36), such as is observed for mutant GCO_E129Q_ (*SI Appendix*, Fig. S7*A*).

Light activation converts GCO_E102Q_ to a species that absorbs primarily with a λ_max_ ~ 380 nm, accompanied with a small amount of 480-nm absorbing species. The latter suggests the possible presence of other photointermediates, the nature of which are not clear at the present time. Subsequent acidification (1 min after bleaching) reveals some A_440 nm_ species (*SI Appendix*, Fig. S7*B*). This indicates that some protonated retinal Schiff base is still present, and shows that the GCO_E102Q_ mutant has slower MII decay/retinal dissociation than the GCO_WT_ receptor. We also tested the effect of three other substitutions at E102 inGCO: GCO_E102D_, GCO_E102V_, and GCO_E102M_. Compared to GCO_WT_, these mutants all showed blue-shifted absorbance λmax (*SI Appendix*, Fig. S7*C*), albeit to different extents.

#### Active-state decay rates measured for Rho_WT_, GCO_WT_, and mutant GCO_E102Q_.

To better define and compare the active-state/MII decay of the samples, we monitored their increase in Trp fluorescence that occurs during loss of all-*trans*-retinal (ATR) from the active receptor (37). An example is shown in Fig. 3*F*, which shows that at 9 °C, the retinal release for activated Rho_WT_ has a t_1/2_ of ~2,188 s. In contrast, active GCO_WT_ decays ~100× faster (t_1/2_ ~ 18 s). Interestingly, the E102Q mutation (GCO_E102Q_) slows the decay of the active state by ~8× (t_1/2_ ~ 139 s). The results are consistent with the retention of an acid-sensitive chromophore described above (Fig. 3*E*). The other substitutions at E102 (noted above) also slowed active-state decay, but to different extents (*SI Appendix*, Fig. S7*D*). These mutants were not studied further, as they either expressed and purified poorly (GCO_E102M_ and GCO_E102V_), or showed only minor differences to GCO_WT_ (GCO_E102D_).

### Gt Activation Ability Measurements for Rho_WT_, GCO_WT_, and Mutant GCO_E102Q_.

The functional ability of the samples (i.e., their ability to activate G protein) was measured using transducin (Gt) purified from native bovine retina. Gt is well characterized and is known to couple with both rhodopsin and cone opsins (38, 39). Because our Gt activation assay measures the increase in Trp fluorescence that occurs in the Gα subunit when it exchanges GDP for GTPγS (40), we first optimized our conditions (6) to ensure the Gt activation signal was not obscured by the Trp fluorescence increase that also accompanies retinal release.

The G protein activation results are shown in Fig. 3 *G*–*J*. Importantly, these results can be directly compared, as they were measured under identical conditions and protein concentrations and were carried out in triplicate, first using a 20× and then a 40× molar excess of Gt to receptor. The results confirm that the large Trp fluorescence increases are caused by Gt activation and nucleotide exchange, as they only occur when Gt and GTPγS are present, and they are much larger than the signal due to retinal release from activated receptor alone. Note: When measured in the absence of Gt, the GCO_WT_ and GCO_E102Q_ samples show higher levels of Trp fluorescence than Rho_WT_ because they decay faster, and thus fully release their retinal during the span of the measurement (10 min). In contrast, the slower decay of Rho_WT_ results in less release of retinal during the measurement, and thus less overall Trp fluorescence increase.

Interestingly, all three samples showed roughly similar apparent initial rates of Trp fluorescence increase upon light activation. This suggests that, qualitatively, they may have similar rates of activation (Fig. 3*J*), although more refined analysis of the type previously described (6, 8) is necessary to definitively compare their initial rates. However, the samples clearly showed differences in their *overall* ability to activate the *total* pool of Gt. At both Gt concentrations tested, GCO_WT_ showed significantly lower activation of the total pool of Gt than Rho_WT_. In contrast, mutant GCO_E102Q_ exhibited a similar total Gt activation as Rho_WT_ (Fig. 3*J*).

These results correlate with the different active-state stability values shown in Fig. 3*F*, and qualitatively support a role for the rapid decay of GCO in setting the overall activation number (6, 8–11). Together, the results suggest that the main reason GCO_WT_ activates fewer G proteins is because it decays so rapidly—slowing this decay with a mutation that increases agonist dwell time (GCO_E102Q_) results in increased total Gt activation.

### Eyring Analysis Suggests That Entropic Differences Underlie the Faster Retinal Release from in Active-State GCO.

To gain insight into what causes the different MII decay rates, we assessed the underlying energetics involved. This involved measuring retinal release rates over a range of temperatures and subjecting the data to Arrhenius and Eyring analysis.

**Fig. 4.**
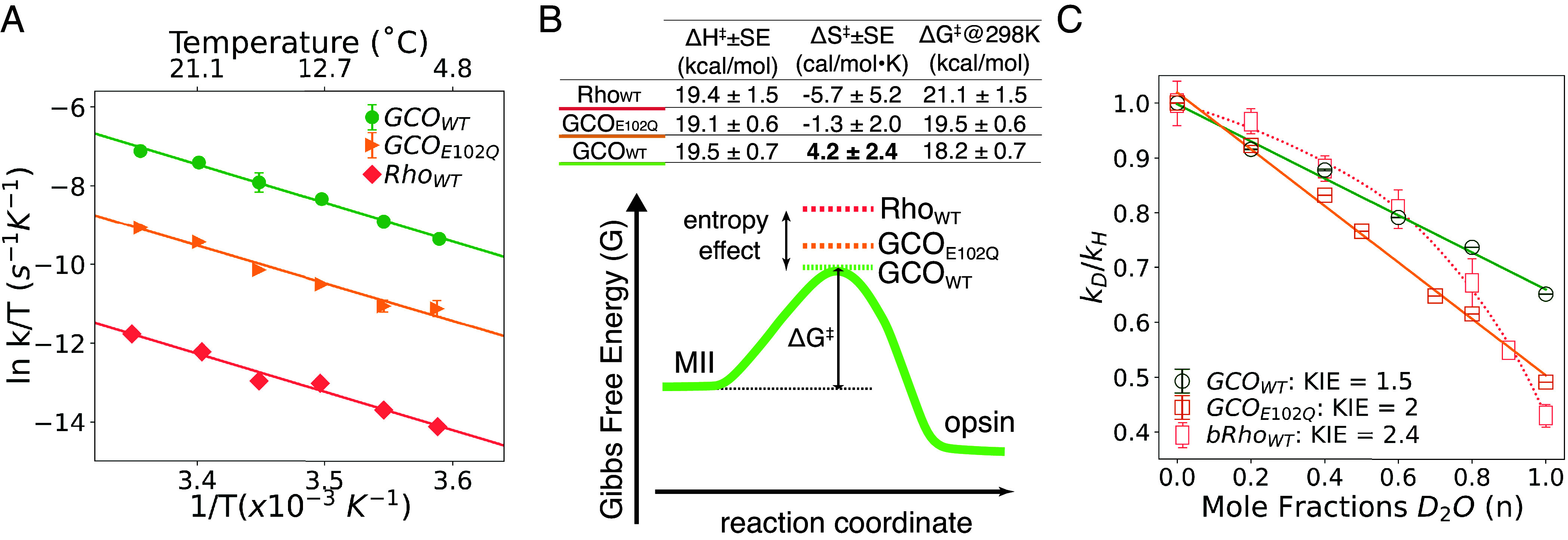
Residue E102^2.53^ plays a key role in the active-state decay of GCO. (*A*) Eyring analysis of MII decays for purified human Rho_WT_, GCO_WT_, and GCO_E102Q_, measured as described in Fig. 3*F* and calculated as described in the Methods. Note that the plots show different rates of decay for the samples, but similar slopes. Data points represent averages of multiple measurements (n = 3) except for rhodopsin (n = 1), with corresponding error bars shown. (*B*, *Top*) Table summarizing the Eyring analysis of the data in (*A*). The results show that all three samples have similar activation enthalpies (ΔH^‡^), but different activation entropies (ΔS^‡^) and activation barrier (∆G^‡^ = ΔH^‡^ − TΔS^‡^, T = 298 K) values for the transition state. Note that the transition state entropy (ΔS^‡^) for GCO_WT_ is positive and larger than Rho_WT_ and GCO_E102Q_. (*Bottom*) Reaction coordinate diagram comparing transition state energies of GCO_WT_, GCO_E102Q_, Rho_WT_. The diagram illustrates how the active-state decay rates for the receptors differ primarily due to differences in entropy, ΔS^‡^. (*C*) Proton inventory analysis measuring the effect of D_2_O on the retinal release rates for GCO_WT_ and GCO_E102Q_. Note that the GCO_WT_ and GCO_E102Q_ data can be fit with linear regression, suggesting a single site of proton transfer is involved in their Schiff base hydrolysis. This differs from bRho_WT_ (data from ref. 41), which produces a curved plot and suggests a multisite proton transfer is required.

Our results show that at all temperatures, the MII decay rates exhibit the following order: GCO_WT_ > GCO_E102Q_ > Rho_WT_. Intriguingly, Arrhenius analysis of the data (*SI Appendix*, Fig. S8) yield plots of nearly identical slopes and thus similar activation energies (E_a_). These E_a_ values are consistent with those previously obtained for rhodopsin (41–44) and suggest similar energetics for the Schiff base hydrolysis reaction. We also carried out Eyring analysis of the same data (Fig. 4 *A* and *B*). The Eyring analysis shows that the samples all have a similar transition-state (TS) **enthalpy** for hydrolysis (ΔH^‡^), but differences in **entropy** of the TS for the reaction (ΔS^‡^). The implications of this finding are examined further in the *Discussion*.

#### Effect of D_2_O on active-state decay of GCO_WT_ and GCO_E102Q_.

We looked for possible differences in a proton-transfer event involving water (H_2_O) during retinal Schiff base hydrolysis by comparing the retinal release rates for GCO_WT_ and GCO_E102Q_ in water (H_2_O) and deuterium isotope (D_2_O). *SI Appendix*, Fig. S9 shows that at 9 °C, the t_½_ for MII decay for GCO_WT_ is slower in D_2_O (H_2_O: 23.3 ± 6.0E-3 s, D_2_O: 35.6 ± 4.8E-3 s, n = 3), indicating a solvent isotope effect of ~1.54 ± 2.00E-4; whereas mutant GCO_E102Q_ showed an even larger D_2_O effect (H_2_O: 131.6 ± 2.8E-2 s, D_2_O: 268.3 ± 2.7E-2 s, n = 3 for both), yielding a solvent isotope effect of ~2.04 ± 1.00E-4 (Fig. 4*C* and *SI Appendix*, Fig. S9).

#### D_2_O (proton inventory) analysis shows that a proton-transfer event during retinal Schiff base hydrolysis of GCO_WT_ may involve residue E102.

We expanded these studies to include a proton inventory analysis, carrying out these measurements using different molar amounts of D_2_O. The results (Fig. 4*C*) show both samples exhibit a linear relationship for the effect of D_2_O on retinal release, with GCO_E102Q_ showing an even steeper slope compared to GCO_WT_ (Fig. 4*C*). These linear relationships are in contrast to our previous studies of bRho_WT_ (41), which showed a markedly nonlinear relationship in its proton inventory analysis. The implications of these findings are discussed below.

## Discussion

Our goal was to determine whether GCO_WT_ exhibits lower levels of G protein activation because it binds G proteins differently than Rho_WT_, or because it decays faster once activated. We also sought to identify factors that cause this rapid active-state decay and retinal release. Together, our results can be used to directly compare both possibilities in a structural and mechanistic context and assess the relative contribution of each. (It should be noted that these mechanisms are not mutually exclusive).

### GCO_WT_ and bRho_WT_ Have Very Similar Interactions with the Gα Subunit.

We find that GCO_WT_ and bRho_WT_ share most of the same interactions with the Gα C-terminus (Gα-CT, also called the α5 helix). As shown in Fig. 1, critical interactions are observed, such as a terminal Phe residue in the Gα-CT, which loops back to form a hook-like structure and nests into the hydrophobic cleft in the receptor formed upon outward movement of TM5 and TM6 (Fig. 1 *B* and *C*). This “hall-mark” interaction is observed in all GPCR-G protein complexes (30, 45). Further examples of similarities in how GCO and various Rhos interact with the Gα-CT/α5 helix are shown in *SI Appendix*, Fig. S5. Of note, we also observe a possible water density coordinated by Y239^5.58^, Y322^7.53^, and backbone carbonyl of L144^3.43^ (*SI Appendix*, Fig. S10*B*). Interestingly, the presence of this bound water is conserved in the active structures of class A GPCRs (46), and mutation of either conserved tyrosine residue reduces G protein activation in rhodopsin (47–49).

Based on these findings, we speculate that GCO_WT_ unlikely exhibits lower levels of G protein activation because of deviations in how it interacts with the G protein. However, confirming this proposal would require more extensive studies. Moreover, our structure does not consider agonist and/or G protein off-rates—critical factors involved in activation. To address this latter possibility, we carried out the biochemical and functional studies discussed below.

### Slowing GCO Active-State Decay Increases Its G Protein Activation to Similar Levels as Rho.

Fig. 3 clearly shows that GCO_WT_ decays much faster than Rho_WT_ and exhibits a reduced overall ability to activate a pool of Gt. Slowing this decay by introducing mutation E102Q (GCO_E102Q_) results in G protein activation levels that are about the same as Rho_WT_. Together, these results confirm that rapid loss of the retinal agonist from activated GCO_WT_ is the main reason for its reduced ability to activate G proteins, by reducing the amount of signaling receptors present.

What causes the faster retinal release from active-state GCO_WT_? Our structural comparisons provide clues.

Unique aspects of the retinal binding pocket contribute to the faster decay of active-state GCO. Comparing active-state structures identifies several factors that likely cause the lower stability of activated GCO_WT_: Specifically, GCO_WT_ has 1) a bigger overall retinal cavity, 2) a larger access channel for H_2_O to enter this cavity, 3) a larger potential “exit” hole the retinal (that could also provide an additional water access route), and 4) a key residue, E102, located nearby that appears to facilitate the retinal Schiff base hydrolysis. The ramifications of these features are discussed in more detail below.

**Fig. 5.**
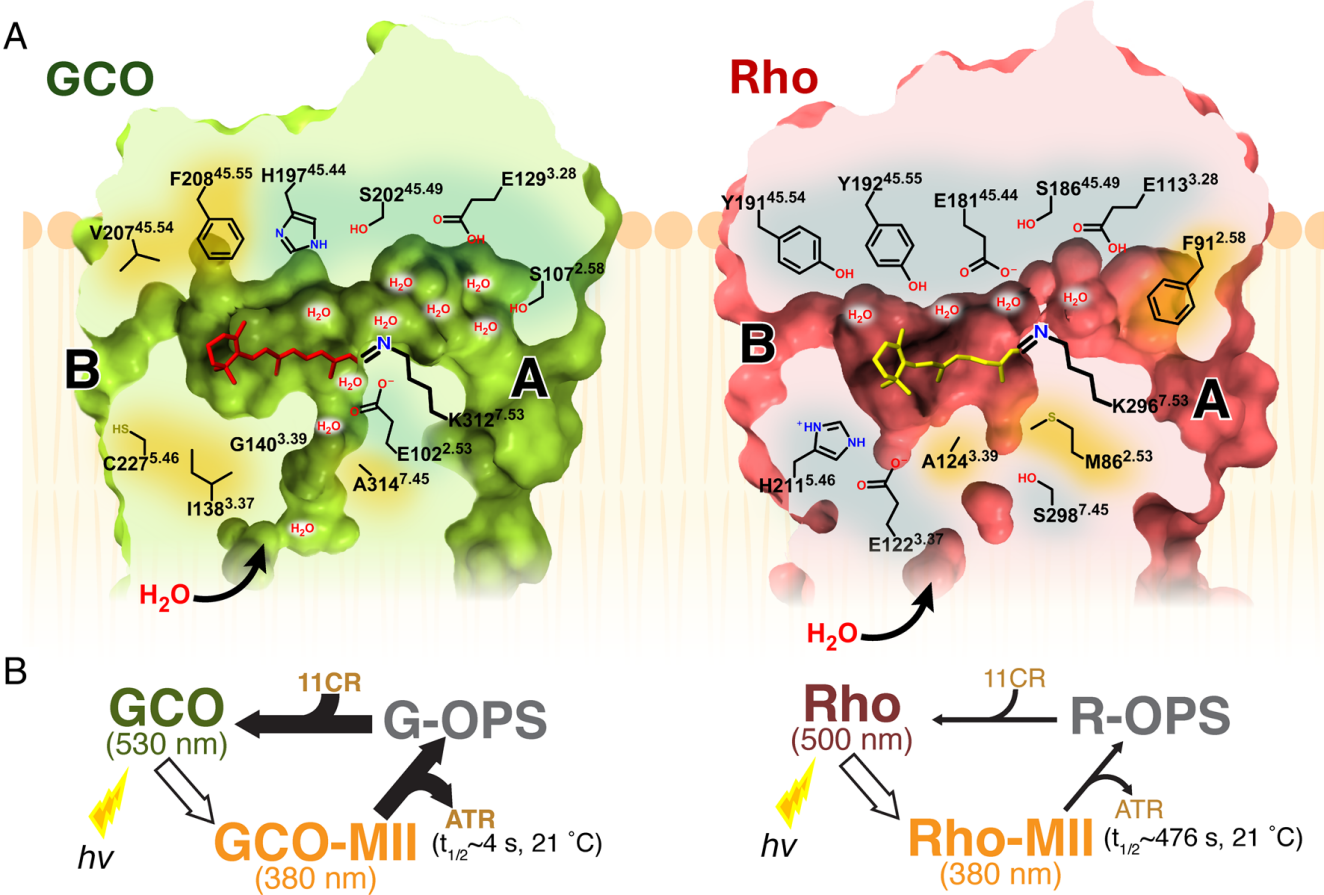
Cartoon highlighting key differences between the active-state structures of GCO and Rho, and their functional implications. (*A*, *Top*) Cartoons derived from sliced-views of the active-state structures of GCO_WT_ and bRho_WT_ (PDB:6OYA). The images illustrate how several factors contribute to the faster hydrolysis and release of retinal from GCO_WT_, including i) a larger retinal binding cavity that can accommodate more H_2_O, ii) greater access to this cavity due to a larger water channel, iii) a larger opening (Hole A) that could provide a faster escape route for hydrolyzed ATR, and iv) polar residues concentrated exclusively near the retinal Schiff base in GCO_WT_. (*B*) (*Bottom*) Schematics comparing the retinal binding and release “lifecycle” for GCO and Rho. As indicated, light converts both receptors to the active MII state, but active-state GCO (GCO-MII) decays rapidly to inactive opsin (Ops), releasing the ATR agonists within seconds, ~100× faster than Rho-MII. Under bright light conditions, this fast ATR release helps to attenuate active GCO-MII signaling and is necessary for the receptor to rapidly reset (rebind 11CR).

1. A bigger retinal cavity that can accommodate more total water molecules. In the active-state structures, the internal cavity in GCO_WT_ is ~1.5× bigger than in bRho_WT_ (~1454.3 Å^3^ vs. 842.4 Å^3^, respectively). These values were calculated by first using KVfinder in ChimeraX (23, 50) to determine the volume of the retinal-free cavity, from which the volume of a retinal ligand (295.3 Å^3^) was then subtracted (*SI Appendix*, Fig. S11). This difference means that in the active state, GCO_WT_ can potentially accommodate up to ~2× more internal H_2_O molecules than Rho_WT_ (12) (~48 H_2_O vs. ~28 H_2_O, respectively, estimated based on density of water being 1 g/mL, which corresponds to 3.35E-2 H_2_O/Å^3^). We also tested whether GCO_WT_ can accommodate more internal waters in silico, by carrying out short molecular dynamics (MD) simulations in YASARA (n = 10, each with a different random seed). In agreement with above, the results show that on average, GCO_WT_ can accommodate ~ 52 ± 3 internal H_2_O molecules, and Rho_WT_ 29 ± 3 internal H_2_O molecules (*SI Appendix*, Fig. S12). These observations are consistent with other experimental results suggesting GCO_WT_ can accommodate more waters in the retinal cavity, some of which could participate in Schiff base hydrolysis (31).

2. A larger access channel for H_2_O to enter the retinal cavity. Both experimental and MD simulation studies indicate that upon Rho activation, water molecules can diffuse into a pore that connects the retinal binding cavity with the solvent-exposed cytoplasmic cleft where the Gα-CT binds (51–54). Our GCO_WT_ structure shows that this pore is larger and more accessible than it is in bRho_WT_ (Fig. 5 and *SI Appendix*, Fig. S11), and thus could potentially allow even more water to gain access to the retinal binding cavity.

3. A larger Hole A that could provide a better “escape route” for retinal and potentially act as second route for water access to the Schiff base. Hole B, the opening between TM5/6 (Fig. 2*A*) is the likely access route for 11CR binding to Rho_WT_ (55). While Hole B is similar in size for both bRho_WT_ and GCO_WT_ (*SI Appendix*, Fig. S13), Hole A (between TM1 and TM7) is significantly larger in GCO_WT_. Importantly, this opening lies right next to the retinal Schiff base linkage (Fig. 2), where it could provide another access route for and accommodation of more water.

4. A unique residue, E102, that facilitates retinal release from active GCO_WT_. A glutamate at position 2.53 is unique to GCO and RCO (Fig. 3*B* and *SI Appendix*, Fig. S6). While E102 is not the primary counterion in dark-state GCO_WT_ (Fig. 3*E*), it could still act as a “secondary counterion” that plays a role in other photoactivated states, similar to residue E181 in rhodopsin (25–27). Both our current structure and previous MD simulations show that E102 is in close proximity to K312 in the active state, suggesting other possible counterion functions are still possible (56). However, one thing is certain - E102 facilitates retinal release from active GCO_WT_ (Figs. 3*F* and 4*A*). Our kinetic analysis (discussed below) provides clues on how it could do this.

A lower entropic barrier for retinal release results in faster active-state GCO decay. Eyring analysis of our retinal release data (Fig. 4*A*) shows similar changes in transition-state **enthalpies** (ΔH^‡^) for both GCO_WT_ and Rho_WT_. However, the change in **entropy** (ΔS^‡^) is greater in GCO_WT_, resulting in differences in the activation barrier (∆G^‡^). This suggests a larger ΔS^‡^ is the main reason GCO_WT_ decays faster (since chemical reaction rates are influenced by the free energy of activation, i.e., ΔG^‡^ = ΔH^‡^ − TΔS^‡^).

What could cause this larger ΔS^‡^ in GCO_WT_? One possibility is that the reaction transition state (TS) for GCO_WT_ is more disordered and statistically favorable than it is in Rho_WT_. The larger retinal binding cavity in GCO_WT_ would facilitate this, by providing more room for Schiff base hydrolysis intermediates and/or enabling more orientations for internal attacking waters. Alternatively, the TS in both receptors could be similarly ordered, but the *ground* state in GCO_WT_ could be more ordered *before* TS hydrolysis than it is in Rho_WT_.

Comparing the Eyring analyses of GCO_WT_ and GCO_E102Q_ further highlights the role entropic factors play in facilitating retinal release. We had assumed E102 would directly affect the hydrolysis chemistry, because it is so close to the Schiff base (Fig. 3*A*). Instead, while neutralizing E102 (GCO_E102Q_) slows retinal release, there is no change in the TS enthalpy (ΔH^‡^)—only the TS entropy ΔS^‡^ is reduced (Fig. 4 *A* and *B*). This is in contrast to mutations in bRho that slow MII decay, where both the TS enthalpy (ΔH^‡^) and the entropy (ΔS^‡^) are changed (*SI Appendix*, Fig. S14).

How then can E102 contribute to the larger ΔS^‡^ seen in GCO_WT_? One possibility is that E102 precoordinates water molecules *before* Schiff base hydrolysis, and releasing these constraints could yield the positive ΔS^‡^ seen for GCO_WT_. This could also explain why a smaller ΔS^‡^ is seen for GCO_E102Q_, since E102Q might have fewer constraints to be removed. Intriguingly, our structure suggests that E102 could interact with an Asp residue (D99^2.50^) through a bridging water (*SI Appendix*, Fig. S10*A*). This is noteworthy, as this Asp on TM2 is highly conserved in GPCRs, and is known to interact with the highly conserved NPXXY motif (57). Weakening this (potential) interaction in GCO_WT_ during receptor activation could also cause an observed increased entropy (ΔS^‡^) of the transition state.

One caveat—our measurements only detect retinal release from the receptor. While Schiff-base hydrolysis must happen before release can occur, our analysis cannot formally define how much each process contributes to the overall reaction, or how the inherent affinity for the retinal ligand, without the Schiff base, might differ, as has been proposed for blue cone opsin (BCO) by Knox and coworkers (44). Thus, other factors could contribute to the positive TS entropy (ΔS^‡^) we observe for GCO_WT_, such as a nearby larger exit hole that would lend itself to faster ATR escape, and also act as a route for internally bound water molecules to flow through as the active receptor conformation collapses to the inactive state could account for the observed positive entropy. The latter point would be consistent with the recently proposed hypothesis that “water movement into (“sponge”) and through (“conduit”) photoactivated opsins” can be the cause of the elongation and swelling of cone and rod cell outer segments observed upon light activation (58).

Finally, our studies using D_2_O to measure kinetic isotope effects (KIE) clearly show water is involved in the retinal release process, but it is difficult to interpret their implications in detail. Both GCO_WT_ and GCO_E102Q_ show a linear proton inventory plot (Fig. 4*C*), indicating a single site of proton transfer is involved in their Schiff base hydrolysis (59, 60). In contrast, bRho_WT_ exhibits a curved proton inventory plot, indicating a multisite proton transfer is involved (41).

The KIE is larger in GCO_E102Q_ than in GCO_WT_, which might indicate E102 facilitates proton transfer and makes Schiff base hydrolysis process less burdened (59), but it is not clear how this squares with the Eyring analysis that shows the TS enthalpy (ΔH^‡^) is the same for both. Nonetheless, a recent FTIR study (31) has shown that E102 is involved in hydrogen bonding alterations during early steps of light activation, which could be relevant to the difference in KIE we see between GCO_WT_ and GCO_E102Q_.

The active-state structure of GCO_WT_ is poised for fast release of ATR. Fig. 5 summarizes how the features in active-state GCO_WT_ could work together and result in faster ATR release than Rho_WT_.

GCO has a larger cavity surrounding ATR, a larger channel that connects to this cavity, and a concentration of polar or potential negatively charged residues (like E102) near the Schiff base. Together, these features would facilitate water entering the receptor and gaining access to the Schiff base for hydrolysis. The larger Hole A next to the retinal attachment could speed up active-state decay, by providing a bigger escape route that increases the probability of ATR escaping before it potentially re-forms a Schiff base, and by providing an additional route of access for water that could participate in Schiff base hydrolysis.

In contrast, active-state Rho has a smaller internal cavity, smaller opening near the Schiff base and polar residues spread out around the ATR. This constrained arrangement could result in a more extended H-bonding network around the binding pocket but fewer overall incoming waters. Moreover, a smaller Hole A opening near the Schiff base in Rho would slow ATR release compared to GCO.

Functional consequence of faster active-state decay in GCO_WT_. Rapid retinal release and decay of active GCO_WT_ appear to be facilitated by structural features inherent to the protein. This leads to a key question—why would nature conserve these features, like E102, that decrease the stability of the active, signaling state?

The likely answer is that the rapid decay of active cone opsins is necessary for their function and is required to enable cone dominated vision under bright light conditions. This hypothesis (61–63) proposes that rapid decay of active-state cone opsins could serve several crucial roles. One role would be to limit the accumulation of photoactivated, signaling cone opsin receptors that could occur if the signal attenuation machinery becomes overwhelmed (64). Moreover, a fast release of the ATR would be necessary for a cone opsin to quickly “reset” by binding new 11CR, in this way increasing the frequency that it can detect photons under bright light conditions (11). Finally, since cone opsins have a higher rate of 11CR thermal isomerization, rapidly releasing the ATR thus produced could also help decrease the concomitant background signaling (65).

The features identified here that shorten how long GCO_WT_ can signal facilitates its function under bright light, in contrast to dim-light photoreceptor rhodopsin where a longer activation would be more advantageous (66–68). How our findings compare to the other human visual pigments, red cone opsin (RCO) and blue cone opsin (BCO), remains to be seen.

## Materials and Methods

### Buffers, Cloning, Mutagenesis, Transfection, and Purification of Human Green Cone Opsin Samples.

Full-length wild-type human rhodopsin (Rho_WT_) and wild-type human green cone opsins (with the last nine residues replaced with the 1D4 epitope TETSQVAPA) (69) were synthesized and cloned into the PMT4 plasmid, with any subsequent mutagenesis carried out using overlap extension PCR (70). Proteins were expressed by transient transfection in COS-1 cells. To enhance receptor stability, 10 µM β-ionone was added to the medium for cells expressing the GCO_WT_ and mutant GCOs (32). Transfected cells were harvested after ~50 to 54 h, regenerated with 11CR and purified using 1D4 immunoaffinity as previously described for rhodopsin (20, 71). See *SI Appendix, Materials and Methods* for full details.

### Expression and Purification of Receptors and G Protein Complex.

GCO_WT_-G protein complexes were prepared by coexpressing the GCO_WT_ with venus-mini-Gα_ist_, Gβ_1-1D4ET_ and Gγ_2_ in COS-1 cells using transient transfection using PEI in a 15-cm plate. To enhance complex stability, instead of β-ionone, all-trans-retinal (ATR, 10 µM) was added to the medium of cells expressing the complex. Transfected cells were harvested after ~50 to 54 h, washed with PBSSC with 0.5 mM PMSF, scraped free from the plates, pelleted, and snap-frozen in liquid nitrogen and stored in the dark at −80 °C until use. Complex was purified with 1D4 immunoaffinity in the presence of 5 µM ATR, then scFv16 and additional 20 µM ATR added to stabilize the complex before further purification using size exclusion chromatography. See *SI Appendix, Materials and Methods* for full details.

### CryoEM Sample Preparation and Data Collection.

GCO_WT_/G protein complexes were blotted onto glow-discharged Quantifoil holey carbon grid (R1.2/1.3, Au, 300 mesh) using Vitrobot. Grids were imaged using a 200 keV Glacios cryo electron microscope with Falcon4i direct electron detector and Selectris imaging filter (10 eV slit width). A total of 4,190 movies were recorded at pixel size of 0.918 Å/pix and total dose of 37.2 e^−^/pix^2^. See *SI Appendix, Materials and Methods* for full details.

### Cryo-EM Image Analysis and Model Building.

Cryo-EM data processing was performed using CryoSPARC (72). After iterative two- and three-dimensional (2D and 3D) classifications, a final stack of 639,873 particles was selected for final refinement producing a map with resolutions reported in *SI Appendix*, Table S1. AlphaFold model of active-state human green cone opsin (73) and heterotrimeric G proteins and scFv16 from PDB:7P00 (74) were used as the starting reference models. Complex model was refined using phenix real space refine (75) and ROSETTA (76) and manually adjusted using COOT (77). See *SI Appendix, Materials and Methods* for full details.

### Spectral Characterization and G Protein Activation Assays.

UV-Visible measurements used a Shimadzu UV-1601 with temperature-controlled cell holder. Light activation was carried out using a 150 W fiber optic illuminator with 500 nm long-pass filter. The fluorescent measurements of retinal release and Gt activation were carried out using a modified PTI steady-state fluorometer with LED excitation sources (35, 74). See *SI Appendix, Materials and Methods* for full details.

## Supplementary Material

Appendix 01 (PDF)

## Data Availability

The 3D cryo-EM density map for the GCO_WT_/G protein complex has been deposited in the Electron Microscopy Data Bank under accession code EMD-72798. The atomic coordinates for the corresponding structural model of the complex has been deposited in the Protein Data Bank under accession code 9YDA. Previously published data were used for this work (41). All other study data are included in the manuscript and/or *SI Appendix*.
