# Supplementary material for: Structure of human green cone opsin yields insights into mechanisms underlying the rapid decay of its active, signaling state
Source: Proc Natl Acad Sci U S A. 2025 Dec 2;122(49):e2516318122. doi: 10.1073/pnas.2516318122 (PMC12704717; doi:10.1073/pnas.2516318122)
Supplement: Supplementary file 1 — Appendix 01 (PDF) [file pnas.2516318122.sapp.pdf]

Supporting Information for:

Structure of human green cone opsin yields insights into mechanisms underlying the rapid decay of its active, signaling state.

Weekie Yao[1], Jonathan F. Fay[2], David L. Farrens[1*]

[1]Department of Chemical Physiology and Biochemistry, Oregon Health & Science University, Portland, Oregon 97239.

[2] Department of Biochemistry and Molecular Biology, University of Maryland Baltimore, Baltimore, Maryland, 21201

* Corresponding author: David L. Farrens

Email: farrensd@ohsu.edu

This PDF file includes:

- SI Appendix
- Figures S1 to S14
- Tables S1
- SI References

Supporting Information (SI) - Appendix

Material and Methods

Buffers.

11CR was generously provided by C. Makino, Boston University, School of Medicine, Department of Physiology & Physiology, MA. The 1D4 peptide (TETSQVAPA) was purchased from GenScript. LMNG was purchased from Anatrace. All other chemicals were purchased from either Sigma-Aldrich, Fisher or GoldBio.

Buffers used were as follows: hypotonic buffer (20 mM HEPES, pH 7, 40 mM NaCl, 0.5 mM PMSF, 5 μg/ml leupeptin), solubilization buffer (50 mM HEPES, pH 7, 250 mM NaCl, 5% (v/v) glycerol, 0.5 mM PMSF, 5 μg/ml leupeptin, 0.6% (w/v) LMNG, 0.06% (w/v) CHS), wash buffer 1 (50 mM HEPES, pH 7, 250 mM NaCl, 5% (v/v) glycerol, 0.01% (w/v) LMNG, 0.001% (w/v) CHS, wash buffer 2 (5 mM MES, pH 6, 40 mM NaCl, 5% (v/v) glycerol, 0.01% (w/v) LMNG, 0.001% (w/v) CHS, buffer A (5 mM MES, pH 6, 140 mM NaCl, 5% (v/v) glycerol, 0.01% (w/v) LMNG, 0.001% (w/v) CHS), transducin activity assay buffer (20 mM HEPES, pH 7, 140 mM NaCl, 5 mM $MgCl_2$, 0.00075% (w/v) LMNG, 0.000075% (w/v) CHS, 1 mM DTT), hypotonic buffer 2 (20 mM MES, pH 6, 50 mM NaCl, 0.5 mM PMSF, 5 μg/ml leupeptin), solubilization buffer 2 (20 mM MES, pH 6, 100 mM NaCl, 5% (v/v) glycerol, 0.5 mM PMSF, 5 μg/ml leupeptin, 0.6% (w/v) LMNG, 0.06% (w/v) CHS), wash buffer 3 (20 mM MES, pH 6, 100 mM NaCl, 5% (v/v) glycerol, 0.01% (w/v) LMNG, 0.001% (w/v) CHS), SEC buffer (20 mM HEPES, 100 mM NaCl, 0.1 mM TCEP, 0.00075% (w/v) LMNG, 0.000075% (w/v) CHS, 0.00025% (w/v) GDN, pH 7).

Mutant Generation.

Single-site mutations were generated by overlap extension PCR in a synthetic green cone opsin (hGCO) gene (the last 9 residues were replaced with the 1D4 epitope TETSQVAPA) and subcloned into the PMT4 expression vector, as previously described (1). For obtaining active state GCO-G protein complex, we used mini-Gα variant lacking the dynamic alpha helical domain for enhanced receptor binding. The mini-Gα construct is modified from the published sequences of mini-$G\alpha_{s/i1}$48 (2): venus fluorescent protein is added to the N-terminal linked with GSGSG linker for fluorescent detection of the purified complex; initial 16 residues of $G\alpha_{i1}$ is added right before the mini-$G\alpha_{s/i1}$48 construct to enable binding of the single-chain variable antibody fragment scFv16; last 10 residues of the mini-$G\alpha_{s/i1}$48 C-terminal tail are replaced with a high affinity variant of transducin C-terminal tail (LEDLKSCGLF,(3)) for enhanced binding with GCO. The modified venus-mini-$G\alpha_{ist}$ construct is inserted into the pcDNA3.1 expression vector. Full length human $G\beta_1$ is tagged with 1D4 epitope on the C-terminal and subcloned into the pcDNA3.1 expression vector. Full length human $G\gamma_2$ is incorporated in the pcDNA3.1 expression vector. Sanger sequencing was used to confirm all constructs. Sanger sequencing was used to confirm all constructs.

Expression and Purification of Visual Pigments.

Expression and purification of all constructs were carried out as previously described, with some modification (4, 5). Briefly, constructs were expressed in COS-1 cells by transient transfection using PEI in 15-cm plate. To enhance receptor stability, 10 μM β-ionone was added to the medium for cells expressing the GCO_{WT} and mutant GCOs (6). Cells expressing GCO_{WT} and mutant GCOs were harvested after ~ 50 hr, whereas cells expressing Rho_{WT} were harvested after 65-70 hr. Cells harvesting involved first washing the plates with PBSSC (containing 0.5 mM PMSF), then scraping the cells in ~2 ml buffer/plate and transferring the solution to a Falcon tube. The opsin samples were regenerated at room temperature in 20 μM 11CR (GCO_{WT} and mutant GCOs: 30 min; Rho_{WT}: 2 hr). After regeneration, cells were pelleted and snap-frozen in liquid nitrogen and stored in the dark at -80 ℃ until use.

Receptor solubilization and purification were carried out in a dark room under dim red light, as follows. Cell pellets were thawed and resuspended in hypotonic buffer (2 ml buffer/plate) supplemented with 10 μM 11CR. Cells were lysed with a Dounce homogenizer with ~ 60 strokes, and then the membranes were pelleted at 160,000 x g for 30 min at 4 ℃. Membranes were solubilized in solubilization buffer (2 ml buffer/plate) supplemented with 10 μM 11CR and nutated at 4

℃ for 90 min. The membrane lysate was then clarified at 160,000 x g for 30 min at 4 ℃ and the supernatant transferred (supplemented with an additional 10 µM 11CR) and incubated with 1D4 antibody beads in batch for 90 min at 4 ℃ with constant nutation. The protein-bound beads were transferred to a column and washed by gravity flow, first with 40 CV (column volume) of wash buffer 1 followed by 10 CV wash buffer 2. Elution from the protein-bound beads was carried out in steps by first rinsing the beads with 1 CV of wash buffer 2 containing 200 µM 1D4 peptide, then incubation with 1 CV of the same buffer for 2.5 hrs to overnight at 4 ℃. The elution incubation was repeated 1 time.

Concentrations of the purified, 11CR regenerated receptors were determined from their respective maximal long-wavelength absorbance (λ_{MAX}), at 500 nm for Rho_{WT}, 530 nm for GCO_{WT}, and 520 nm for GCO_{E102Q}, and a chromophore extinction coefficient (ε) of 40,000 $cm^{-1}M^{-1}$(7). Protein quality (the relative fraction of the purified receptor that was regenerated with chromophore) was determined from the ratio of protein absorbance (A_{280}) and the chromophore absorbance (A_{500} or A_{530}). The ε_{280} values were estimated using ProtParam, ExPasy, yielding ε_{280} for rhodopsin= 64,000 $cm^{-1}M^{-1}$, and ε_{280} for apo GCO: 99,350 $cm^{-1}M^{-1}$. Using this approach, we found the A_{280}/A_{500} for purified hRho is ~ 1.8; and the A_{280}/A_{530} for hGCO was ~ 3.3 (with a theoretical value of ~ 2.8 for the latter).

Absorbance Spectroscopy.

All UV-visible spectra were collected using a Shimadzu UV-1601 with temperature-controlled cell holder (TCC-240A, Shimadzu). For routine dark state and bleached spectra, measurements were taken at room temperature (~ 21 ℃), and sample bleaching was carried out for 15s using a Techni-Quip Corp T-Q/FOI-1 150W fiber optic illuminator with 500 nm long-pass filter. Acid protonation of the Schiff base was performed by the addition of 2 µl of 0.8 N H_2SO_4 to 80 µl of bleached or dark state sample.

Fluorescence Spectroscopy.

All fluorescence was measured using a modified PTI QuantaMaster steady-state fluorometer. In this instrument, the excitation light is generated by an Ocean Optics LLS-295 LED (triggered by the PTI software), then attenuated by a neutral density filter (ND1.7), then directed to the sample though a bifurcated fiber optic cable. Temperature was maintained using a water-jacketed cuvette holder and a VWR 1160 water bath and constantly monitored with an Omega Thermistor probe (44004). A typical measurement used 0.5 µM protein in 60 µl volume in buffer A. Trp emission was monitored through a monochromator set at 330 nm (emission slits 20-nm), and an additional 340 nm band-pass filter to reject light used in photo-bleaching from reaching the emission detector.

Retinal release rates measured by time-resolved steady-state fluorescence.

Retinal release after light activation were monitored as previously described (8), using the fluorescence instrumental setup described above. Measurements used repeated excitation cycles (2 sec on then 3 sec off) from the 295 nm LED, and emission monitored at 330 nm. At the start of each experiment, a baseline was measured for ~ 3 min to ensure stability. At "t=0", samples were then photo-activated with light directed through a fiber optic cable and focusing lens for 2 sec. Photo-activation of GCO_{WT} and mutant GCO_{E102Q} used a Fostec ACE illuminator (150 W) filtered through a 500-nm long-pass filter. Fluorescence was monitored until a plateau in the signal. Rates of retinal release were determined by fitting the fluorescence data to a mono-exponential rise to maximum equation: $F(t) = F_\infty\left(1 - e^{-kt}\right) + C$. For Eyring analysis, the rates obtained from a series of retinal release measurements (carried out between 5-25 ℃) were fit to the Eyring equation: $\ln\left(\frac{k}{T}\right) = -\frac{\Delta H^\ddagger}{R}\frac{1}{T} + \ln\left(\frac{k_B}{h}\right) + \frac{\Delta S^\ddagger}{R}$ to determine the activation enthalpy ($\Delta H^\ddagger$) and entropy ($\Delta S^\ddagger$) for retinal release (R, k_B, h are the gas constant, the Boltzmann constant and the Plank constant, respectively).

Transducin (Gt) Activation Monitored by Tryptophan fluorescence.

Activation of Gt by Rho_{WT}, GCO_{WT} and mutant GCO_{E102Q} was monitored as described previously (9), using the same modified PTI QuantaMaster steady-state fluorometer setup described above for retinal release measurements. In brief, visual pigment (15 nM) was added to a reaction mixture containing 300/600 nM Gt and 5/10 µM GTPγS in 400 µl of transducin activity assay buffer in the dark. Fluorescence measurements were carried out at 9 ℃. After a stable baseline was achieved (~ 3 min), samples were photo-activated using a short pulse (2 sec) of light from the appropriate light

source described above, and fluorescence increase was monitored for 900 sec. Gt activation rates were estimated from the first 30 s after photo-activation, using the slopes of the initial fluorescence increase determined from linear regression analysis ("linregress" function in SciPy.stats Python package). The resulting changes of fluorescence were also fit to monoexponential rise to maximum equation: $F(t) = F_\infty(1 - e^{-kt})$ to obtain total increase in fluorescence (F_∞).

Proton Inventory Studies on MII Decay/Retinal Release Rates.

Proton inventory studies on the effect of D_2O on retinal release rates were carried out using slight modifications to our previous approach (10). Samples in D_2O were prepared by first purifying receptors as described above in H_2O, then while still bound to the 1D4 column, washed 1X and eluted in buffers containing D_2O. Proton Inventory measurements used Buffer A prepared in D_2O or H_2O, then combined in various proportions to give different mole fractions (n) of D_2O ranging from 0-1.0. The MII decay rate was monitored as described above at 9 ℃, using the range of different mole fractions of D_2O. Data were analyzed by plotting the ratio of k_D/k_H versus the mole fraction of D_2O, where k_D is the rate in the molar fraction of D_2O and k_H is the rate in 100% H_2O.

Expression and purification of scFv16. The Bac-to-Bac Baculovirus Expression System (Invitrogen) was used in Spodoptera frugiperda Sf9 insect cells to express scFv16 with a His tag attached to the C-terminus. Supernatant containing secreted scFv16 was harvested after 4 to 5 days post infection, when the cell viability was less than 10%. The supernatant from the Sf9 growth as then adjusted to a pH of 7.8 by addition of Tris powder and subsequently chelating agents were added (1 mM NiCl2 and 5mM CaCl2). After centrifugation, the supernatant was then incubated with excess Ni-NTA resin at 4 °C overnight. The beads were washed 15–20 times the bead volume with washing buffer (20 mM HEPES pH 7.5, 500 mM NaCl, 10 mM imidazole). Proteins were eluted with elution buffer (20 mM HEPES pH 7.5, 100 mM NaCl,250 mM imidazole). The eluted samples were then concentrated using a 10-kDa molecular weight cutoff concentrator.

Expression and purification of GCO-G protein complexes for cryo-EM analysis. To assemble GCO-G protein complex, GCO was co-expressed with venus-mini-$G\alpha_{ist}$, $G\beta_{1\text{-}1D4ET}$ and $G\gamma_2$ in COS-1 cells by transient transfection using PEI in 15-cm plate. To enhance complex stability, all trans-retinal (10 µM) was added to the medium of cells expressing the complex. Transfected cells were harvested after ~ 50-54 hr, washed with PBSSC with 0.5 mM PMSF, scraped free from the plates, pelleted and snap-frozen in liquid nitrogen and stored in the dark at -80 ℃ until use.

To initiate complex formation, cell pellets were thawed and resuspended in hypotonic buffer 2 (1.2 ml buffer/plate of cells) supplemented with 20 µM ATR. Cells were lysed with a Dounce homogenizer with ~ 60 strokes and incubated in the dark at 4 ℃ for overnight. To solubilize the GCO-G protein complex, additional NaCl, glycerol and detergent were added to the overnight incubation to match the composition of solubilization buffer 2 (final 2 ml buffer/plate of cell). Additional ATR was also added to the mixture to reach final concentration of 20 µM. The cell lysate was nutated in the dark for 1 hr at 4 ℃ and then clarified at 160,000 x g for 30 min at 4 ℃. The supernatant was transferred (supplemented with another 20 µM ATR) and incubated with 1D4 antibody beads in batch for 90 min at 4 ℃ with constant nutation. The protein-bound beads were then transferred to a column and then washed by gravity flow with 40 CV (column volume) wash buffer 3 supplemented with 5 µM ATR. Elution from the protein-bound beads was carried out in steps by the addition of 1 CV (column volume) of wash buffer 3 with 800 µM 1D4 peptide and 5 µM ATR, then incubation for 2.5 hrs to overnight at 4 ℃, and the process repeated 3 times. Immunoaffinity purified complex was pooled, concentrated with a 100 kDa molecular weight cut-off Amicon Ultra concentrator and then incubated with scFv16 (1.5 molar excess) in the dark at 4 ℃ for 1 hr. The protein complex was further purified using a Superdex 200 Increase 10/300 gel filtration column in SEC buffer at flowrate of 0.5 ml/min. Excess ATR (about 20 µM) was added to the sample before injection onto the gel filtration column. Elution from the column was monitored with absorbance at 280 nm. Fractions containing the complex were pooled, concentrated to about 2-5 mg/ml and sent for cryo-EM analysis.

Cryo-EM data collection and processing. The GCO_{WT}-G protein sample (3.5 µl) was load onto glow-discharged Quantifoil R1.2/1.3 Au300 holey carbon grids, blotted for 3 to 4.5 seconds at 4 ℃ and 100% humidity, and flash-frozen using liquid ethane/propane (40.1%/59.9%) mixture. Grid preparation was done using a Vitrobot mark IV (ThermoScientific). The grids were then transferred to a 200 keV Glacios cryo electron microscope with a Falcon4i direct electron detector and

Selectris imaging filter with a slit width of 10 eV for single-particle cryo-EM data acquisition. Movies were semi-automatically recorded using SerialEM (11, 12). A total of 4,190 movies were recorded at a physical pixel size of 0.918 Å/pixel, a defocus range of -0.2 to -2.1 microns and a total dose of 37.2 e^-/pix^2. Raw movies were processed and manually curated using CryoSPARC (13). Blob particle picker and two-dimensional (2D) classification were used to generate templates for subsequent template particle picking. Curated particles from template picking were combined with selected particles from blob picking and 2D classification.

A subset of these particles was used to train Topaz particle picker to repick particles (14). Curated particles picked with blob, template and Topaz particle picker were combined and subjected to 2D and/or 3D classifications. A final stack of 639,873 particles was selected for final refinement producing a map with resolutions reported in Table S1. Additional map sharpening was performed using deepEMhancer (15).

Model building and refinement. Active-state model of GCO_{WT} generated from AlphaFold (16) was placed into the EM map density using COOT and ChimeraX (17, 18). The mini-$G\alpha_{ist}$ protein hetero trimer and scFv16 was grafted into this model using 7P00 as the primary template for input (19). The residues of the C-terminal of mini-$G\alpha_{ist}$ were mutated in ChimeraX to match this study.

The resultant model then subjected to several iterative rounds of ROSETTA (20) and phenix real space refine (21). The ligands ATR was fetched from monomer library in COOT and manually placed in the cryoEM density using COOT and refined using COOT to generate the final models.

Structure comparison. All structural comparisons between rhodopsin and GCO were performed with 'Matchmaker' function in ChimeraX. All alignments were done with reference to the receptor unless stated specifically. RMSD was calculated with 'Matchmaker' function or 'RMSD' command selecting Cα atoms of specific residues for calculation after alignment with 'Matchmaker'. For RMSD calculations for the overall intracellular region, residues 49-84,124-161,217-259,297-320 in rhodopsin and residues 65-100,140-177,233-275,313-336 in GCO were used ; for RMSD calculations of the overall extracellular region, residues 21-48,85-123,162-216,260-296 in rhodopsin and residues 37-64,101-139,178-232,276-312 in GCO were used. For RMSD calculations for each helix, residues that are in the transmembrane helix were used . Molecular surfaces were shown using default ChimeraX setting. Electrostatic surface potential generated with 'coulombic' command in ChimeraX.

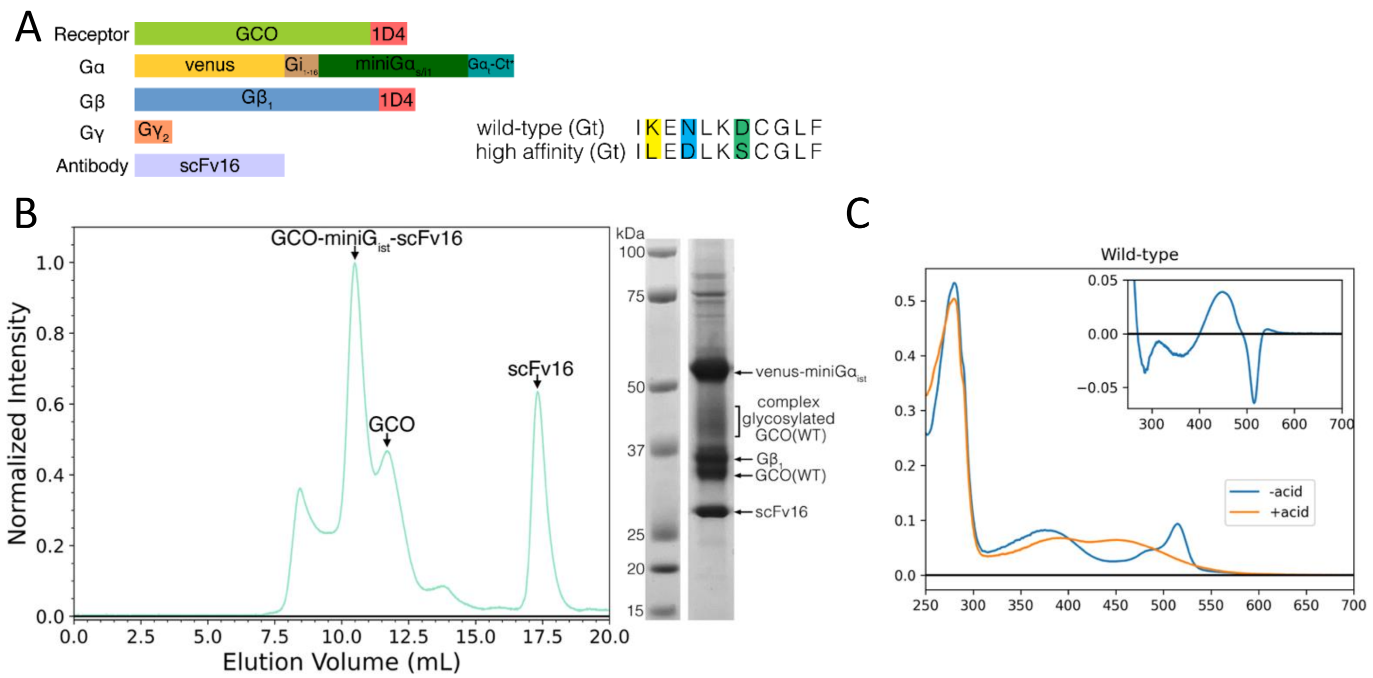

Fig. S1. Cryo-EM sample preparation and analysis for GCO_{WT}-mini-$G\alpha_{ist}$-$G\beta_{1\text{-}1D4ET}$-$G\gamma_2$-scFv16 protein complex.

(A) (left) Design of the GCO_{WT}, mini-$G\alpha_{ist}$, $G\beta_{1\text{-}1D4ET}$, $G\gamma_2$ and scFv16 protein constructs. (right) Sequence comparison of wild-type and high affinity (this study) C-terminal tails of Gtα subunit.

(B) Size exclusion chromatography (SEC) profile and SDS-PAGE of the GCO_{WT}-mini-$G\alpha_{ist}$-$G\beta_{1\text{-}1D4ET}$-$G\gamma_2$-scFv16 protein complex.

(C) UV-vis absorbance spectra of GCO_{WT}-mini-$G\alpha_{ist}$-$G\beta_{1\text{-}1D4ET}$-$G\gamma_2$-scFv16. Spectrum without acid shows two absorbance peaks, one with a λ_{max} at 380 nm and another 515 nm, indicating the presence of retinal chromophore and venus fluorescent protein, respectively. Subsequent acidification of the sample results in a ~440 nm absorbing species, indicating the presence of a protonated retinal-Schiff base linkage. Inset: difference spectrum from measurements pre-/post-acidification. The increase in absorbance at ~ 440 nm indicates the presence of a retinal-Schiff base linkage. The sharp decrease at ~ 520 nm is presumably due to the Venus fluorescent protein tag on the mini-$G\alpha_{ist}$ losing absorbance upon acidification.

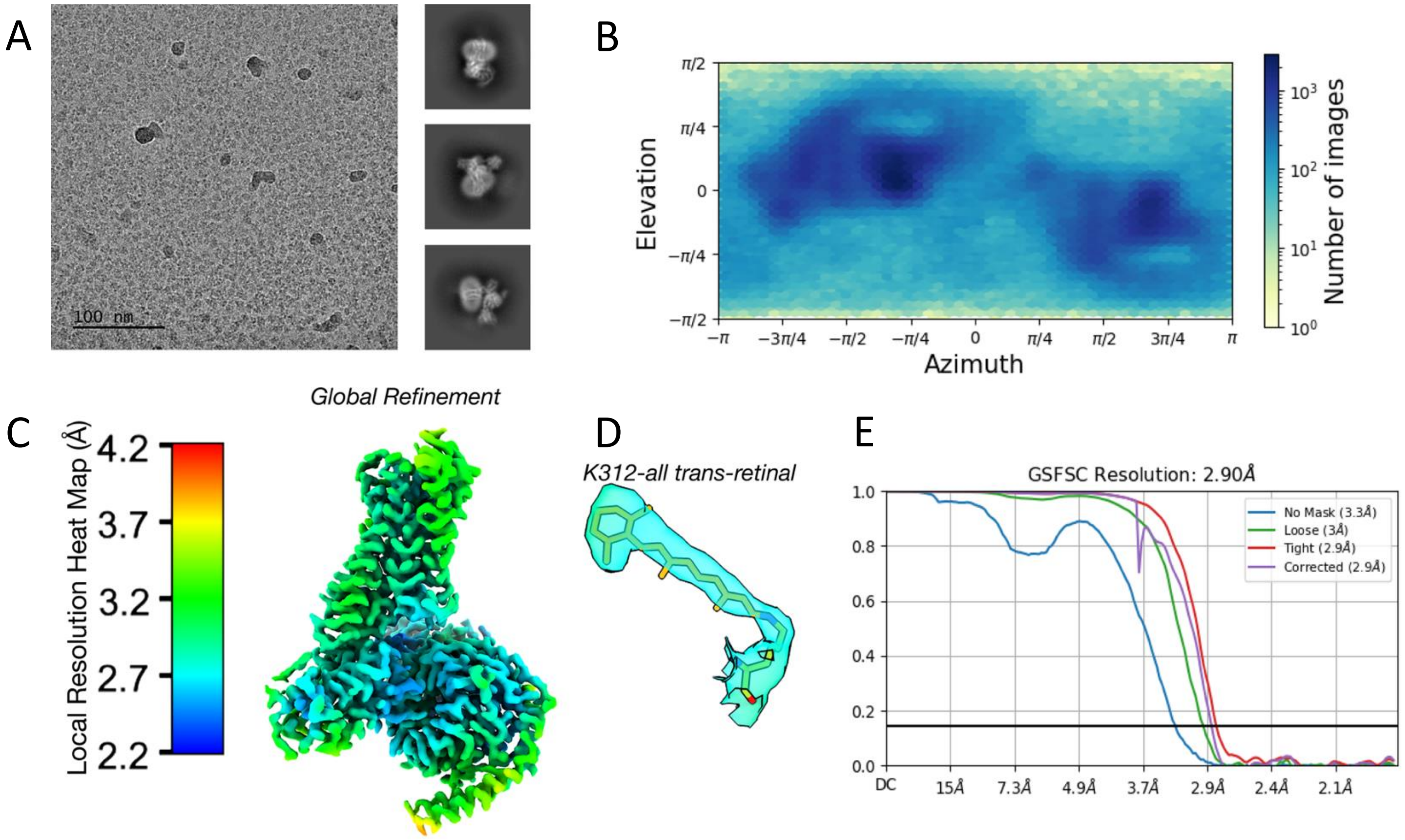

Fig. S2. Cryo-EM data processing of GCOWT / mini-G protein complex.

(A) (left) Representative micrograph of the GCO_{WT}/mini-G protein complex particles described in Fig. S1. (right) Representative 2D classifications.

(B) Orientation distribution heat map.

(C) 3D cryo-EM map of the GCO_{WT} / mini-G protein complex colored with local resolution estimated using the *phenix.local_resolution* function in phenix.

(D) Density of all-trans retinal (ATR) with Schiff base lysine (K312) in receptor, colored using the same local resolution gradient as in (C).

(E) Gold standard Fourier shell correlation (FSC) curve with the estimated resolution at 0.143.

Table S1. Cryo-EM data collection, refinement and validation statistics

Structures	GCO_{WT}-mini-$G\alpha_{ist}$-$G\beta_1$-$G\gamma_2$ ATR (PDB: 9YDA) (EMD-72798)
**Data collection and processing**	
Nominal Magnification	
Voltage (kV)	200
Electron exposure ($e^-/Å^2$)	37.2
Number of movies used	4,190
Defocus mean (SD) µm[1]	1.2 (0.3)
Range	0.2-2.1
Pixel size (Å)	0.918
Symmetry imposed	C1
Initial blob images (no.)	4,064,836
Final particle images (no.)	639,873
Map resolution (Å)	2.90
FSC threshold	0.143
Map resolution range (Å)	2.275-4.689
**Refinement**	
Model resolution (Å) [2]	3.01
FSC threshold	0.5
Map sharpening B factor ($Å^2$)	111.7
Model composition	
Non-hydrogen atoms	9037
Protein residues	1149
Ligands	1
B factors ($Å^2$) (Biso)	
Protein	44.75
Ligand	20.00
CC (volume)	0.81
CC (ligand)	0.69
R.m.s. deviations	
Bond length (Å)	0.006
Bond angle (°)	1.020
Validation	
EMRinger score	4.21
MolProbity score	1.11
Clash score	3.18
Poor rotamer (%)	0.62
Ramachandran Plot	
Favored (%)	98.15
Allowed (%)	1.85
Disallowed (%)	0.0

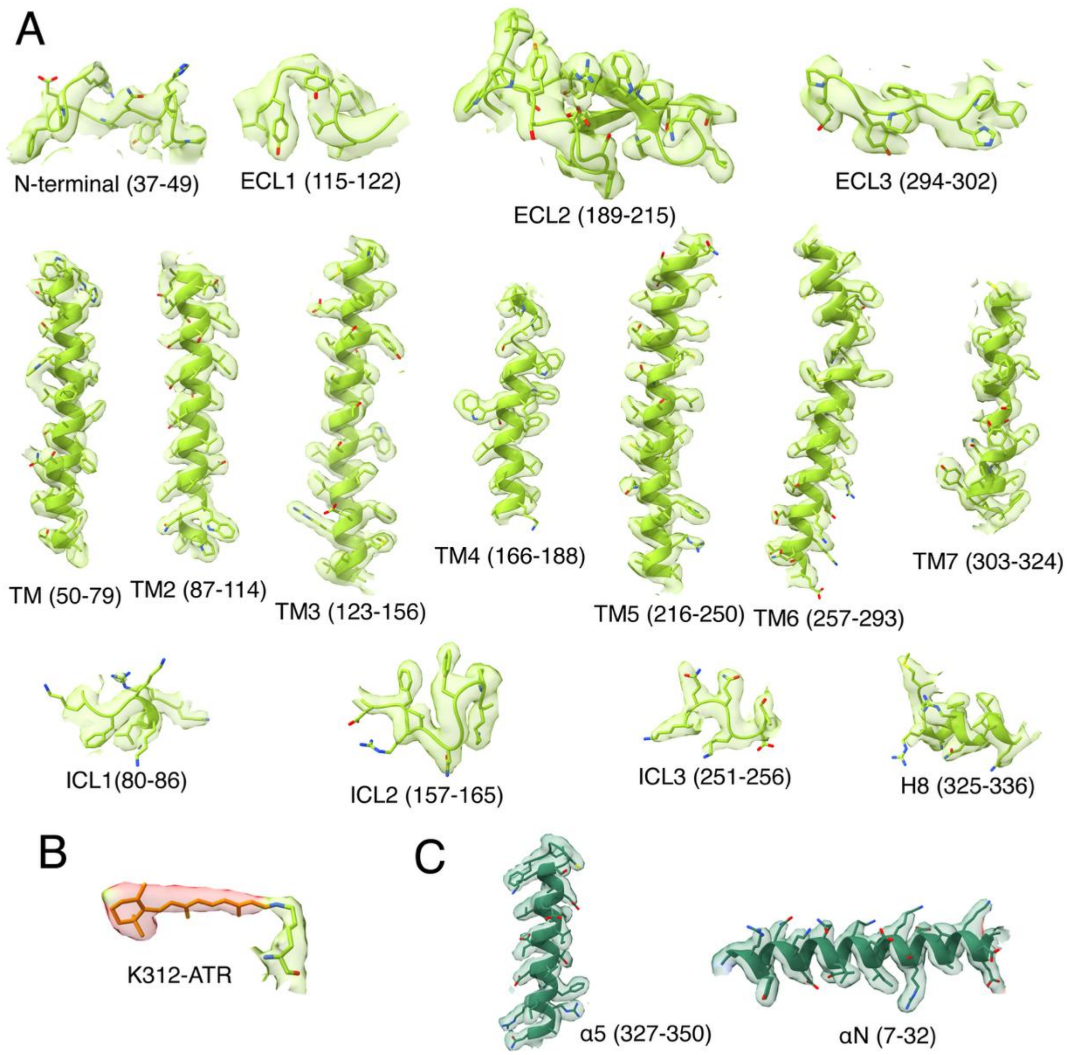

Fig. S3. Representative cryo-EM densities of GCO and mini-Gα_{ist} with fitted models.

(A) Densities of N-terminal, loop regions (ECL1-3 and ICL1-3), TM1-7, and H8 for GCO_{WT}.

(B) Densities of all *trans*-retinal linked to lysine 312.

(C) Densities of selected regions of mini-G protein that interacts with GCO_{WT} (α5 helix: N327-F350, αN helix: A7-A32).

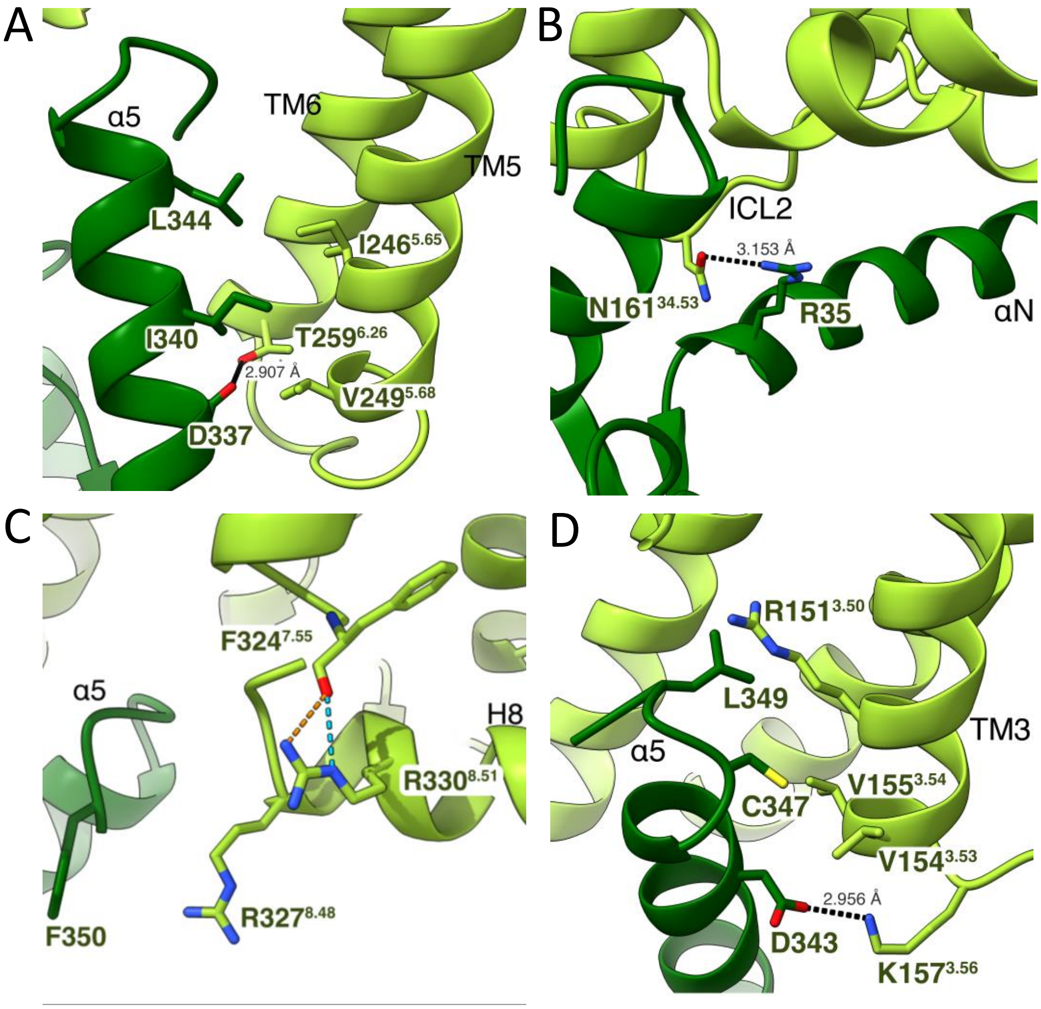

Fig. S4. Interactions between GCO_{WT} and mini-$G\alpha_{ist}$.

A) Hydrophobic interactions between $I246^{5.65}$, $V249^{5.68}$ in TM 5 of GCO_{WT} and L344, I340 in C-terminal helix of mini-$G\alpha_{ist}$. Hydrogen bonding interaction between $T259^{6.26}$ in TM6 of GCO_{WT} and D337 in C-terminal helix of mini-$G\alpha_{ist.}$

B) Hydrogen bonding interaction between $N161^{34.53}$ in ICL2 of GCO_{WT} and R35 in N-terminal helix of mini-$G\alpha_{ist}$.

C) Cation-π interaction between $R327^{8.48}$ in H8 of GCO_{WT} and F350 in C-terminal helix of mini-$G\alpha_{ist}$. $R330^{8.51}$ in H8 of GCO_{WT} appears to form hydrogen bonds with backbone carbonyl of $F324^{7.55}$ in TM7 of GCO_{WT}, interactions that might help to anchor H8 along the plasma membrane. This observation is intriguing, since the R330Q mutation in GCO can cause the visual disease deuteranopia (20). Moreover, GCO mutant R330Q has been shown to have defective transducin binding and reduced visual response in cells (21), which could be due to the absence of R330 to stabilize H8.

D) Hydrophobic interaction between highly conserved arginine in class A GPCR, $R151^{3.50}$ in TM3 in GCO_{WT} and L349 in C-terminal helix of mini-$G\alpha_{ist}$. Hydrophobic interaction among $V154^{3.53}$, $V155^{3.54}$ in TM3 of GCO_{WT} and C347 in C-terminal helix of mini-$G\alpha_{ist}$. Salt bridge between $K157^{3.56}$ in TM3 of GCO_{WT} and D343 (N343D mutation discovered for high affinity rhodopsin binding (3)) in C-terminal helix of mini-$G\alpha_{ist}$.

	TM2	TM3				ICL2		TM5				TM6								TM7	H8			RMSD to
	2.39	3.50	3.53	3.54	3.56	34.55	34.56	5.65	5.68	5.71	5.72	6.23	6.25	6.26	6.28	6.29	6.32	6.33	6.36	7.56	8.47	8.48	8.49	GCO_{WT} (Å)
GCO_{WT}	L88	R151	V154	V155	K157			I246	V249	Q252	Q253		S258	T259		A262	E265	V266	M269		N326	R327		
8Y01	L88	R151	V154	V155	K157			I246	V249	Q252	Q253		S258	T259		A262	E265	V266	M269	M325	N326	R327	Q328	1.380 (17)
6OYA	L72	R135	V138	V139	K141			V230	A233	Q236	Q237		T242	T243		A246	E249	V250	M253		N310	K311	Q312	1.320 (17)
6CMO	L72	R135	V138	V139			F148	V230	A233		Q237	S240	T242	T243		A246	E249	V250	M253	M309	N310	K311	Q312	1.185 (15)
6FUF	L72	R135	V138	V139	K141			V230	A233		Q237		T242	T243	K245	A246	E249	V250			N310	K311	Q312	1.280 (15)
3PQR	L72	R135	V138	V139	K141			V230	A233				T242	T243		A246	E249	V250	M253		N310	K311		1.172 (15)
2X72	L72	R135	V138	V139	K141	R147		V230	A233				T242	T243		A246	E249	V250	M253		N310	K311	Q312	0.999 (15)
4A4M	L72	R135	V138	V139	K141	R147		V230	A233				T242	T243		A246	E249	V250			N310	K311	Q312	1.103 (14)

	α5 helix														RMSD to	Buried surface	Buried surface
	H5.09	H5.12	H5.13	H5.15	H5.16	H5.17	H5.19	H5.20	H5.21	H5.22	H5.23	H5.24	H5.25	H5.26	GCO_{WT}-G (Å)	(tail only) (Å^2)	(whole Gα) (Å^2)
GCO_{WT}	N333	T336	D337		I340	L341	D343	L344		S346	C347	G348	L349	F350		778	947
8Y01	D337		D341	I343	I344	K345	N347	L348	K349	D350	C351	G352	L353	F354	0.471 (24)	888	1015
6OYA	D333		D337		I340	K341	N343	L344	K345	D346	C347	G348	L349	F350	0.527 (24)	833	1072
6CMO			D341		I344	K345	N347	L348	K349	D350	C351	G352	L353	F354	0.527 (24)	693	902
6FUF	D337		D341		I344	A345	N347	L348	R349	G350	C351	G352	L353	Y354	0.463 (24)	788	911
3PQR					I340	L341	N343	L344		D346	V347	G348	L349	F350	0.325 (11)	644	-
2X72					I340	L341	N343	L344		D346	C347	G348	L349	F350	0.344 (11)	690	-
4A4M					I340	L341	N343	L344		D346	C347	G348	L349	F350	0.402 (11)	683	-

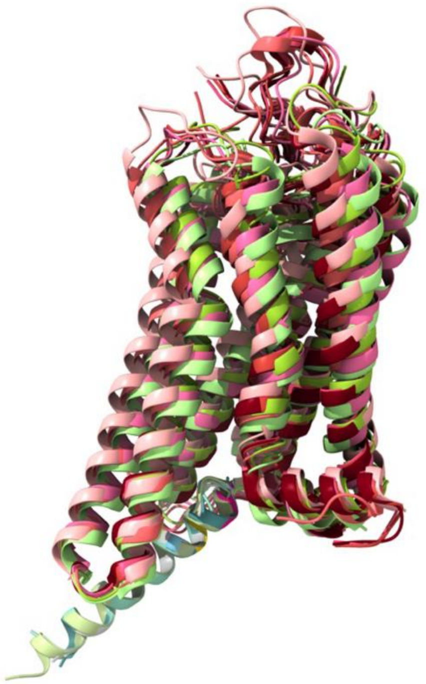

Fig. S5. Comparison of receptor/Gα C-tail interactions for eight structures. Contacting residues between receptor and α5 helix of Gα subunit are assessed using 'Interfaces' function in ChimeraX. Buried surfaces are also calculated in ChimeraX using the same function. RMSD of interacting residues are calculated as follow: alignment of only GαCt, gives RMSD for that region (aligned structures are shown on the left), then manually calculate RMSD of Cα carbon of common tail interacting residues in C-tail aligned position. (PDB: 8Y01 (24), 6OYA (25), 6CMO (26), 6FUF (27), 3PQR (28), 2X72 (29), 4A4M (30).)

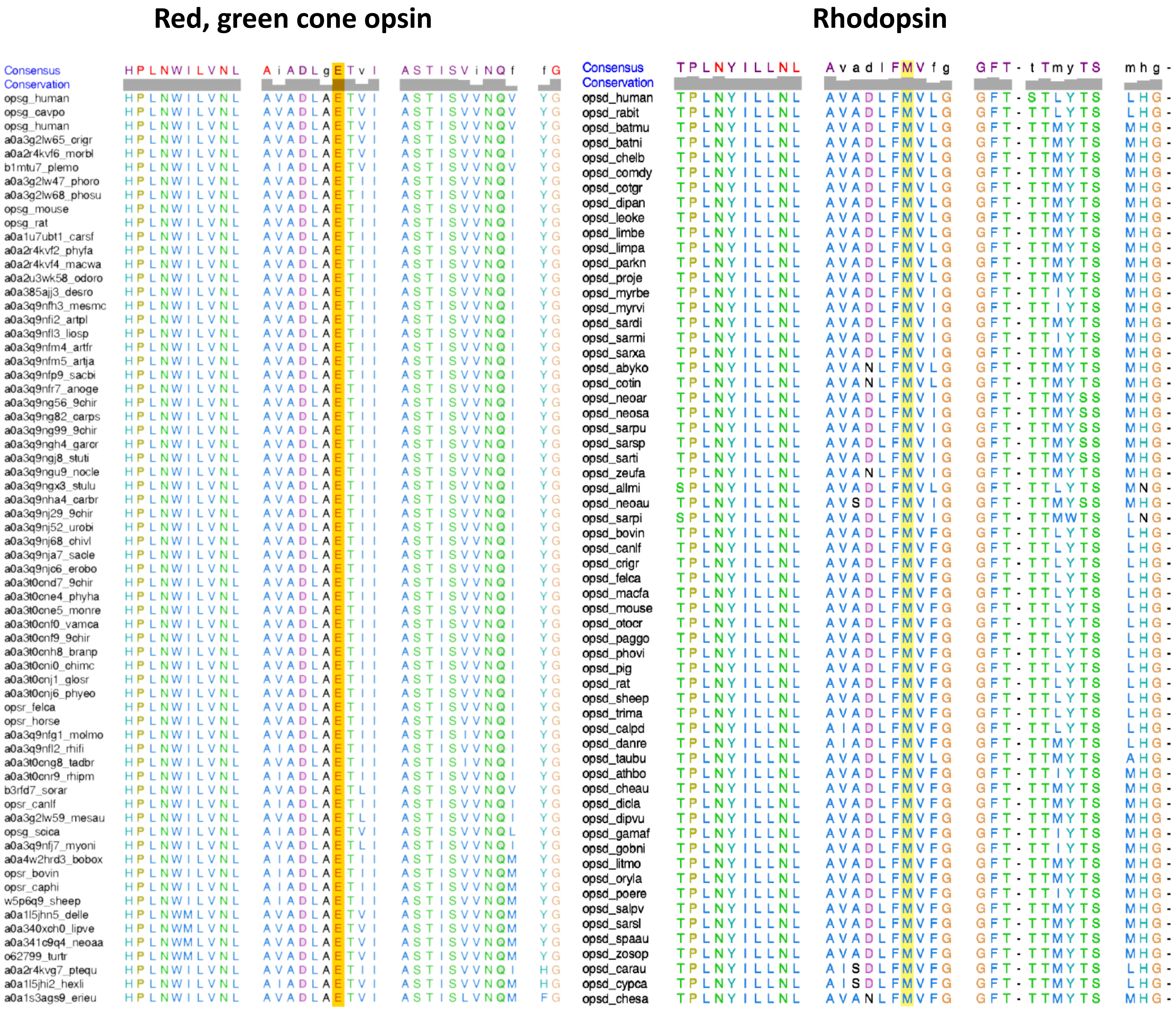

Fig. S6. Sequence alignment of TM2 of medium-, long-wavelength sensitive opsins (left) and rhodopsin (right). The alignments show that $E^{2.53}$ (highlighted in yellow) is highly conserved among all medium- and long-wavelength sensitive opsins. In contrast, in rhodopsins residue $M^{2.53}$ is highly conserved.

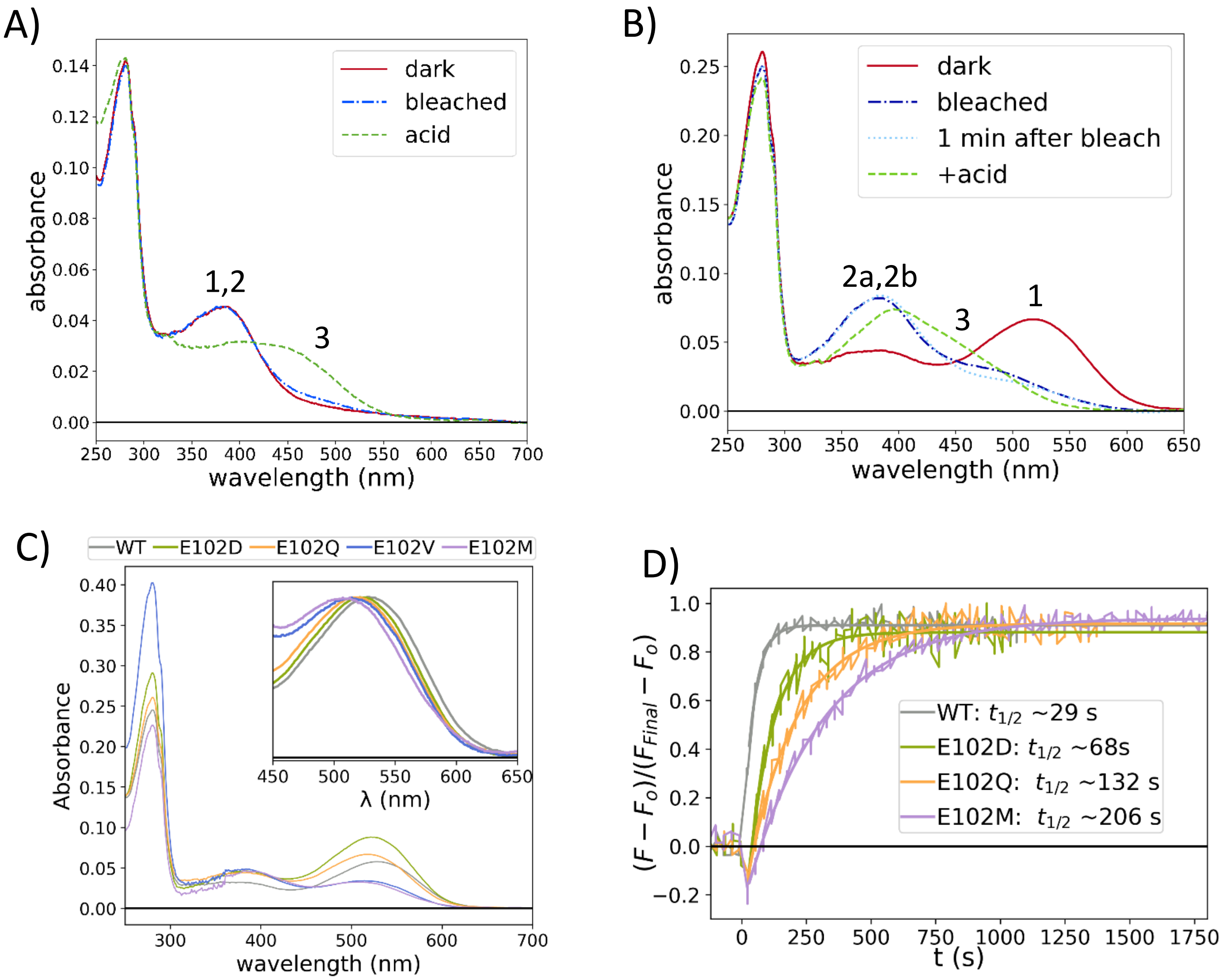

Fig. S7. Characterization of mutant GCO_{E129Q} and GCO E102 mutants. A) Spectra of GCO_{E129Q} were measured in the dark (1) then after activation with UV light (2). The light activated sample was then acidified, yielding a ~440 nm absorbing species, which indicates the retinal-Schiff base linkage is present and can be converted to a protonated form (3). The slight increase in absorbance between 440 and 500 nm after UV illumination (2) could be a MetaIII-like species, similar to that produced upon UV illumination of the rhodopsin equivalent E113Q (31). However, more studies are needed to determine the exact nature of this species. B) Spectra of GCO_{E102Q} were measured in the dark (1) and after light-activation (2a), then measured again 1 minute later (2b), then the sample acidified and measured again (3). The shoulder at ~440 nm in (3) shows protonatable retinal-Schiff base linkages are still present even 1 min after light-activation, indicating GCO_{E02Q} has a slower retinal release rate than GCO_{WT}. C) Comparison of dark-state UV-vis absorbance spectra of GCO mutants containing different substitutions at E102. The substitutions include E102Q (similar size, neutral residue); E102M (rhodopsin equivalent), E102V (~ 30% of all class A GPCRs), and E102D (smaller residue with similar polarity). The spectra yield the following λmax values: GCO_{WT} ~ 530 nm; GCO_{E102D} ~525 nm; GCO_{E102Q} ~520 nm; GCO_{E102V} ~ 514 nm, GCO_{E102}: ~ 513 nm. Inset: Zoomed-in spectra of the mutants normalized to 0.1 for comparison. D) Comparison of retinal release/MII decay measurements for light-activated GCO_{WT} and several of the E102 mutants noted above. Measurements were at ~10 C̊ in low salt buffer (same as buffer A but with 40 mM NaCl instead of 140 mM NaCl).

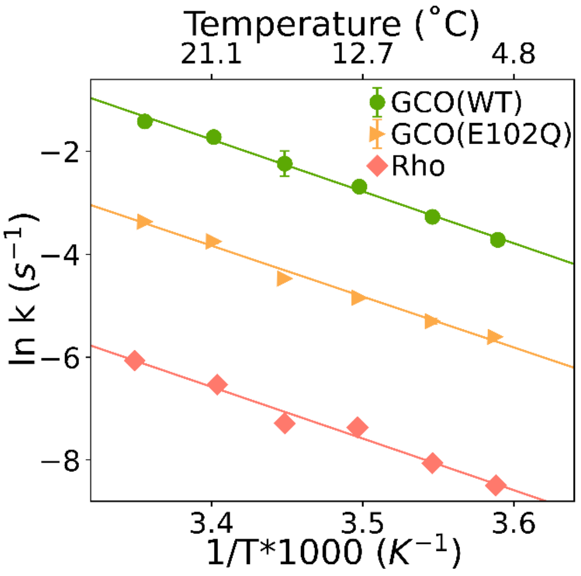

	Ea (kcal/mol)
Rho_{WT}	19.9±4.1
GCO_{WT}	20±1.5
GCO_{E102Q}	19.6±1.2

Fig. S8. Arrhenius analysis of MII decays for Rho_{WT}, GCO_{WT} and GCO_{E102Q}. The plots show different rates of decay for the samples, but similar slopes. Activation energy derived from the slopes are shown in the table on the right.

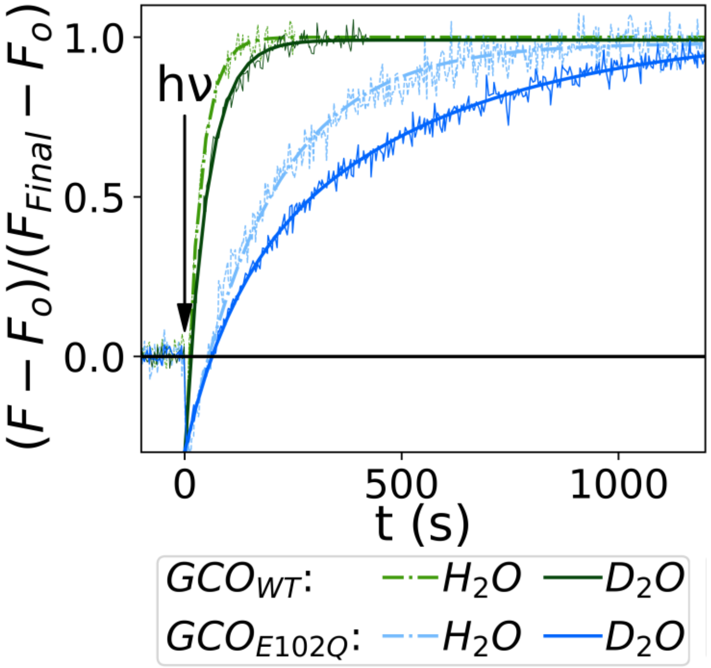

Fig. S9. Deuterium isotope effect on retinal release of GCO_{WT} and GCO_{E102Q}. Comparison of retinal release/MII decay measurements for GCO_{WT} and GCO_{E102Q} in buffer prepared with H_2O (dashed line) or D_2O (solid line).

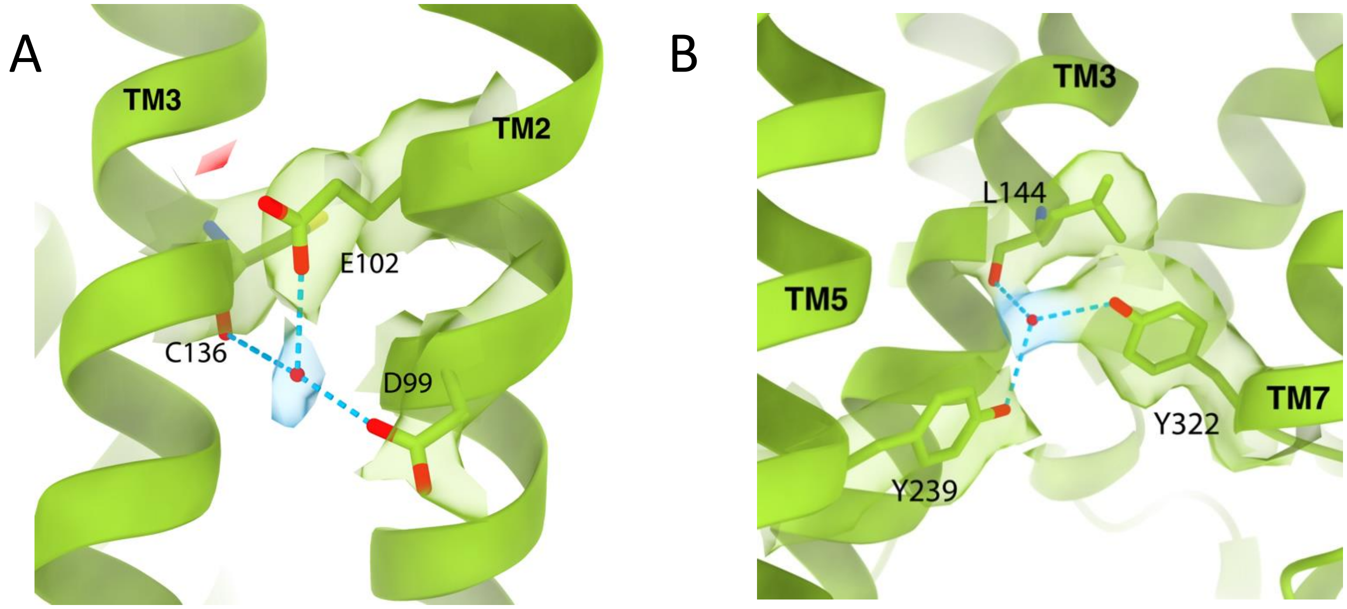

Fig. S10. Potential water densities observed in the cryo-EM map of GCO. (A) Extra density is observed that could be a water molecule coordinated by the E102$^{2.53}$ and D99$^{2.50}$ sidechains and C136$^{3.35}$ backbone carbonyl. (B) Extra density was observed among Y239$^{5.58}$, Y322$^{7.53}$ and L144$^{3.43}$ which corresponds to highly conserved water molecules observed in active structure of class A GPCR among Y$^{5.58}$, Y$^{7.53}$ sidechain and L$^{3.43}$ backbone carbonyl.

Internal cavity

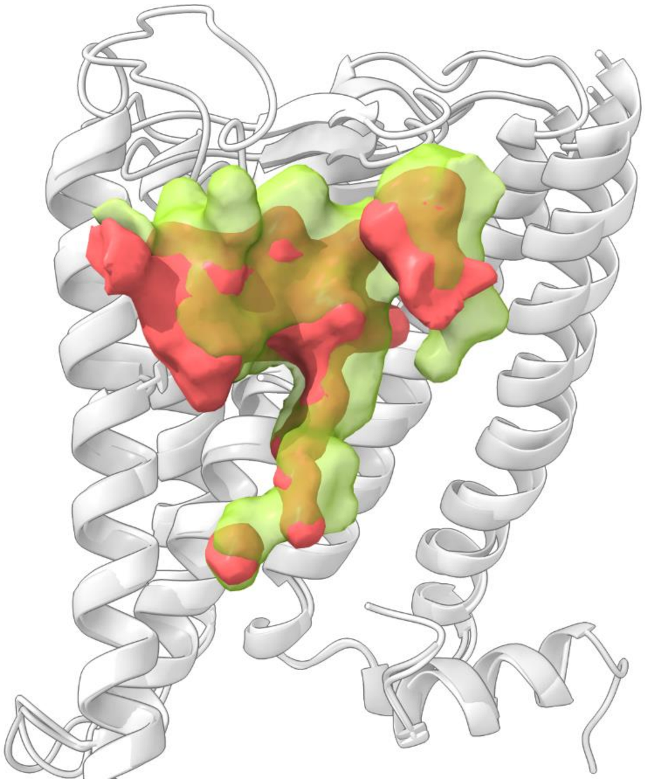

Cytoplasmic face

Fig. S11. Cavities in the active state of GCO_{WT} and Rho_{WT} estimated with pyKVFinder (32) and displayed with ChimeraX (18). TM6 and TM7 are hidden to expose the cavities inside. The internal cavity in GCO_{WT} (green) appears to have an extended cavity that spans across the ligand channel and extends towards the cytoplasmic face. In contrast, the internal cavity in $bRho_{WT}$ (red) appears to have a smaller and truncated ligand channel and a narrower/truncated tunnel that leads to the cytoplasmic face. Cavities volume: GCO_{WT} = 1454.3 Å; Rho_{WT} = 842.37 Å.

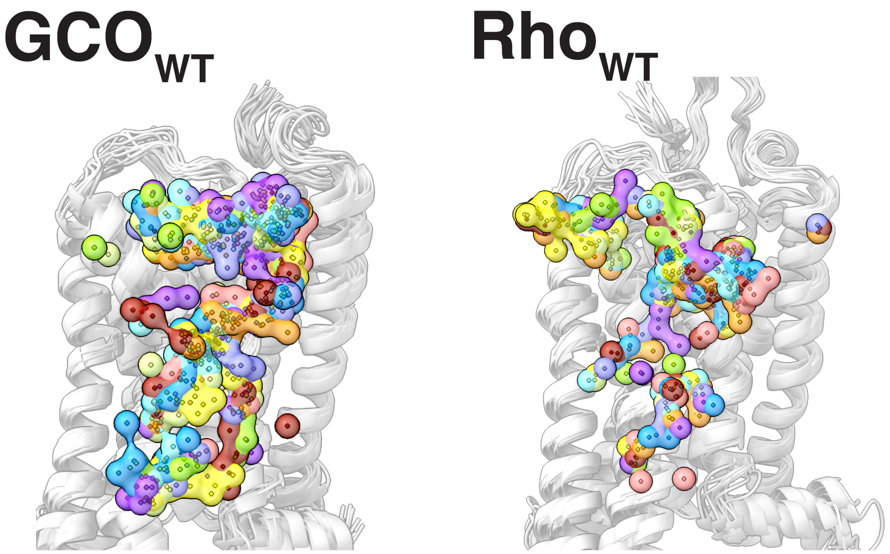

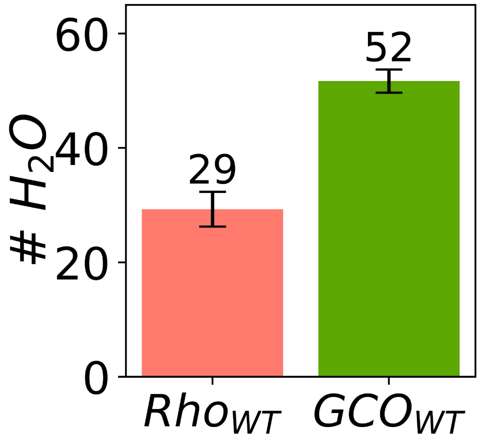

Run #	Rho	GCO
1	24	51
2	35	51
3	31	57
4	22	51
5	26	50
6	34	50
7	30	56
8	28	50
9	31	48
10	32	53

Figure S12. Water cavity comparison of GCO_{WT} and Rho_{WT} based on molecular dynamic (MD) simulation. Ten independent short simulations (with different random seeds) for were run using YASARA and the macro *md_runmembranefast* (33) on the GCO_{WT} receptor, and Rho_{WT} (PDB:3PQR) without post-translational modifications. (top) Receptor structures with internal water molecules from all 10 simulations were shown above (different color indicate different simulation runs). (bottom) Count of internal waters for each simulation were determined by using vmd to identify water molecules within 4 Å of residue atoms (found by cavity estimation). The results were tabulated in the table and the average number of internal water molecules for each receptor with 95% confidence interval was shown in bar graph (GCO: 52±3 (10); Rho: 29±3 (10)). In brief, the simulations were carried out as follows. The initial setup included optimization of the hydrogen bonding network (34) to increase the solute stability, and a pKa prediction to fine-tune the protonation states of protein residues at pH 7.4 (34). NaCl ions were added at physiological concentration of 0.9%, with an excess of either Na or Cl to neutralize the cell. After steepest descent and simulated annealing minimizations to remove clashes, the simulation was run for 250 ps using the AMBER14 force field (35)for the solute, GAFF2 (36)and AM1BCC (37) for ligands and TIP3P for water. Force field parameters for all non-standard residues like the retinal ligand were generated using the AutoSMILES algorithm integrated within the YASARA simulation pipeline (36–39). The cutoff was 8 Å for Van der Waals forces (the default used by AMBER (40)), no cutoff was applied to electrostatic forces (using the Particle Mesh Ewald algorithm, (41)). The equations of motions were integrated with a multiple timestep of 1.25 fs for bonded interactions and 2.5 fs for non-bonded interactions at a temperature of 298 K and a pressure of 1 atm (NPT ensemble) using algorithms described in detail previously (42). The whole simulation cell after 250 ps was then saved as a pdb file for further analysis in vmd.

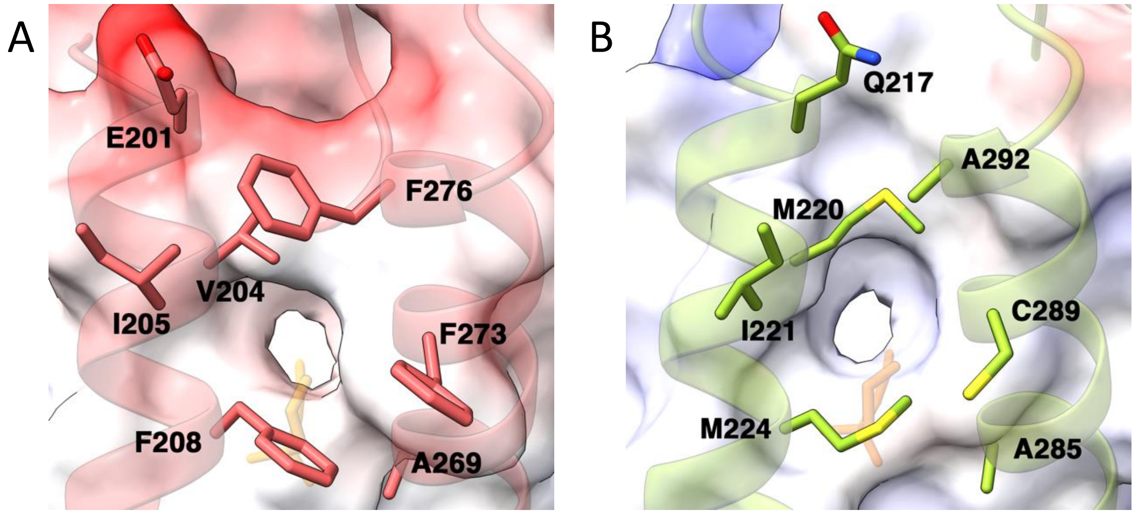

Fig. S13. Comparison of "Hole B" in active-state Rho$_{WT}$ (A) and GCO$_{WT}$ (B). The opening in the active state structures is defined by the indicated residues, with the surface colored with electrostatic surface potential calculated in ChimeraX (18). Despite the differences in the residues, "Hole B" appears to be a similar size in both structures. E201$^{5.36}$ in Rho$_{WT}$ /Q217$^{5.36}$ in GCO$_{WT}$ are shown to due to large difference in local electrostatic surface potential.

A

	ΔH‡ (kcal/mol)	SE	ΔS‡ (cal/mol•K)	SE	TΔS‡ @ 298K (kcal/mol)	ΔG‡ @ 298K (kcal/mol)
**WT**	19.88	0.96	-4.17	3.28	-1.24	21.12
**E181Q**	21.07	0.28	0.87	0.96	0.26	20.81
**Y268F**	20.22	0.55	-1.90	1.87	-0.57	20.78
**Y192F**	14.12	0.31	-22.71	1.05	-6.77	20.88
**S186A**	26.52	1.16	15.25	3.98	4.54	21.98
**E113Q**	39.79	1.42	59.08	4.78	17.61	22.18
**T94I**	24.62	0.75	7.57	2.57	2.26	22.36

B

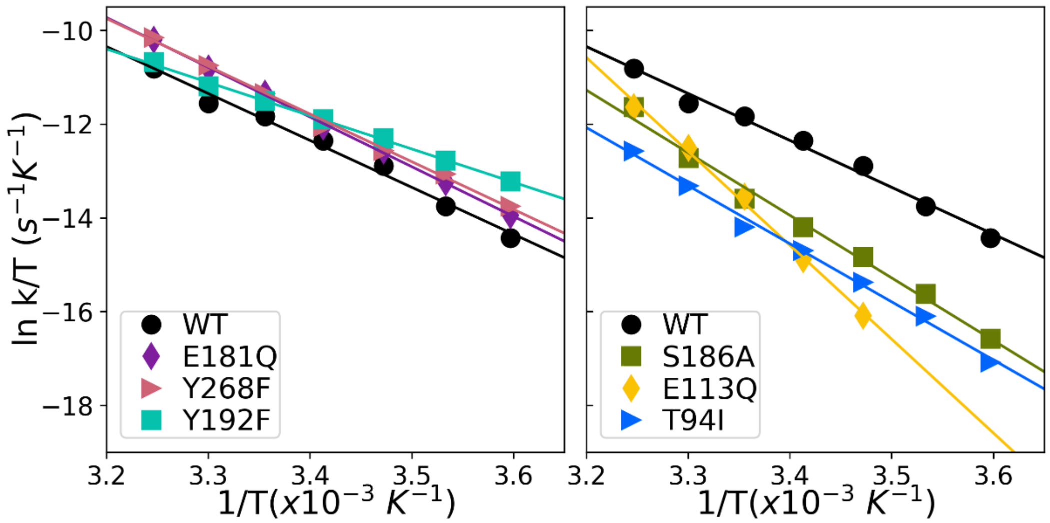

C

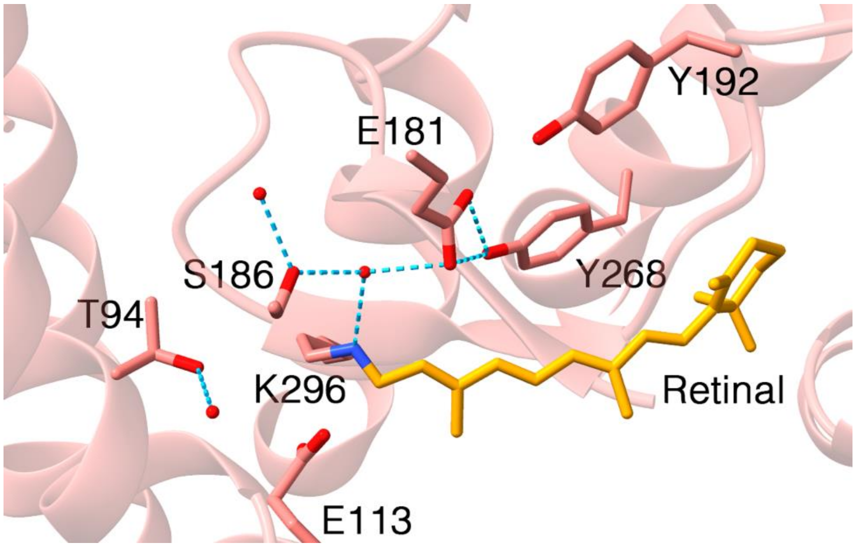

Fig. S14. Eyring analysis of retinal release rates for bovine rhodopsin containing mutations near the retinal Schiff base. For comparison with our results in the present study, we analyzed our previous data that measured retinal release rates for activated wild-type bovine rhodopsin and rhodopsin mutants (43). (A) Table of ΔH‡, ΔS‡ and ΔG‡ values obtained from Eyring analysis of the retinal release rates plotted as a function of temperature in (B). The analysis shows that the mutations in Rho that slow retinal release (S186A, E113Q and T94I) are accompanied with large changes in both enthalpy ΔH‡ and entropy, ΔS‡. (C) Location of the mutated residues in the active structure of rhodopsin (PDB:3PQR).

SI REFERENCES

1. E. Ramon, X. Mao, K. D. Ridge, Studies on the stability of the human cone visual pigments. *Photochem Photobiol* **85**, 509–16 (2009).

2. R. Nehmé, *et al.*, Mini-G proteins: Novel tools for studying GPCRs in their active conformation. *PLoS One* **12**, e0175642 (2017).

3. L. Aris, *et al.*, Structural requirements for the stabilization of metarhodopsin II by the C terminus of the alpha subunit of transducin. *J Biol Chem* **276**, 2333–9 (2001).

4. D. D. Oprian, A. B. Asenjo, N. Lee, S. L. Pelletier, Design, chemical synthesis, and expression of genes for the three human color vision pigments. *Biochemistry* **30**, 11367–11372 (1991).

5. J. M. Janz, D. L. Farrens, Rhodopsin activation exposes a key hydrophobic binding site for the transducin alpha-subunit C terminus. *J Biol Chem* **279**, 29767–73 (2004).

6. S. M. Noorwez, D. A. Ostrov, J. H. McDowell, M. P. Krebs, S. Kaushal, A High-Throughput Screening Method for Small-Molecule Pharmacologic Chaperones of Misfolded Rhodopsin. *Investigative Ophthalmology & Visual Science* **49**, 3224–3230 (2008).

7. P. M. Vissers, W. J. DeGrip, Functional expression of human cone pigments using recombinant baculovirus: compatibility with histidine tagging and evidence for N-glycosylation. *FEBS Lett* **396**, 26–30 (1996).

8. J. M. Janz, D. L. Farrens, Assessing structural elements that influence Schiff base stability: mutants E113Q and D190N destabilize rhodopsin through different mechanisms. *Vision Res* **43**, 2991–3002 (2003).

9. O. P. Ernst, C. Bieri, H. Vogel, K. P. Hofmann, "[32] Intrinsic biophysical monitors of transducin activation: Fluorescence, UV-visible spectroscopy, light scattering, and evanescent field techniques" in *Methods in Enzymology*, Vertebrate Phototransduction and the Visual Cycle, Part A., (Academic Press, 2000), pp. 471–489.

10. J. M. Janz, D. L. Farrens, Role of the retinal hydrogen bond network in rhodopsin Schiff base stability and hydrolysis. *J Biol Chem* **279**, 55886–94 (2004).

11. D. N. Mastronarde, Automated electron microscope tomography using robust prediction of specimen movements. *Journal of Structural Biology* **152**, 36–51 (2005).

12. J. V. Peck, J. F. Fay, J. D. Strauss, High-speed high-resolution data collection on a 200 keV cryo-TEM. *IUCrJ* **9**, 243–252 (2022).

13. A. Punjani, J. L. Rubinstein, D. J. Fleet, M. A. Brubaker, cryoSPARC: algorithms for rapid unsupervised cryo-EM structure determination. *Nat Methods* **14**, 290–296 (2017).

14. T. Bepler, *et al.*, Positive-unlabeled convolutional neural networks for particle picking in cryo-electron micrographs. *Nat Methods* **16**, 1153–1160 (2019).

15. R. Sanchez-Garcia, *et al.*, DeepEMhancer: a deep learning solution for cryo-EM volume post-processing. *Commun Biol* **4**, 1–8 (2021).

16. L. Heo, M. Feig, Multi-state modeling of G-protein coupled receptors at experimental accuracy. *Proteins: Structure, Function, and Bioinformatics* **90**, 1873–1885 (2022).

17. P. Emsley, B. Lohkamp, W. G. Scott, K. Cowtan, Features and development of Coot. *Acta Crystallogr D Biol Crystallogr* **66**, 486–501 (2010).

18. E. C. Meng, *et al.*, UCSF ChimeraX: Tools for structure building and analysis. *Protein Science* **32**, e4792 (2023).

19. C. Thom, *et al.*, Structures of neurokinin 1 receptor in complex with Gq and Gs proteins reveal substance P binding mode and unique activation features. *Science Advances* **7**, eabk2872 (2021).

20. R. Y.-R. Wang, *et al.*, Automated structure refinement of macromolecular assemblies from cryo-EM maps using Rosetta. *eLife* (2016). Available at: https://elifesciences.org/articles/17219 [Accessed 22 April 2025].

21. P. V. Afonine, *et al.*, Real-space refinement in PHENIX for cryo-EM and crystallography. *Acta Crystallogr D Struct Biol* **74**, 531–544 (2018).

22. P. Zhu, *et al.*, Disease mechanisms of X-linked cone dystrophy caused by missense mutations in the red and green cone opsins. *FASEB J* **35**, e21927 (2021).

23. S. Srinivasan, M. A. Fernandez-Sampedro, E. Ramon, P. Garriga, Structural and functional alterations associated with deutan N94K and R330Q mutations of green cone opsin. *Biochim Biophys Acta Mol Basis Dis* **1863**, 1840–1847 (2017).

24. Q. Peng, *et al.*, Cryo-EM structures of human cone visual pigments. [Preprint] (2024). Available at: https://www.biorxiv.org/content/10.1101/2024.01.30.577689v1 [Accessed 12 June 2025].

25. Y. Gao, *et al.*, Structures of the Rhodopsin-Transducin Complex: Insights into G-Protein Activation. *Molecular Cell* **75**, 781-790.e3 (2019).

26. Y. Kang, *et al.*, Cryo-EM structure of human rhodopsin bound to an inhibitory G protein. *Nature* **558**, 553–558 (2018).

27. C.-J. Tsai, *et al.*, Crystal structure of rhodopsin in complex with a mini-Go sheds light on the principles of G protein selectivity. *Sci Adv* **4**, eaat7052 (2018).

28. H. W. Choe, *et al.*, Crystal structure of metarhodopsin II. *Nature* **471**, 651–5 (2011).

29. J. Standfuss, *et al.*, The structural basis of agonist-induced activation in constitutively active rhodopsin. *Nature* **471**, 656–60 (2011).

30. X. Deupi, *et al.*, Stabilized G protein binding site in the structure of constitutively active metarhodopsin-II. *Proc Natl Acad Sci U S A* **109**, 119–24 (2012).

31. J. Standfuss, E. Zaitseva, M. Mahalingam, R. Vogel, Structural impact of the E113Q counterion mutation on the activation and deactivation pathways of the G protein-coupled receptor rhodopsin. *J Mol Biol* **380**, 145–57 (2008).

32. J. V. da S. Guerra, *et al.*, pyKVFinder: an efficient and integrable Python package for biomolecular cavity detection and characterization in data science. *BMC Bioinformatics* **22**, 607 (2021).

33. E. Krieger, G. Vriend, YASARA View - molecular graphics for all devices - from smartphones to workstations. *Bioinformatics* **30**, 2981–2982 (2014).

34. E. Krieger, R. L. Dunbrack, R. W. W. Hooft, B. Krieger, Assignment of protonation states in proteins and ligands: combining pKa prediction with hydrogen bonding network optimization. *Methods Mol Biol* **819**, 405–421 (2012).

35. J. A. Maier, *et al.*, ff14SB: Improving the Accuracy of Protein Side Chain and Backbone Parameters from ff99SB. *J Chem Theory Comput* **11**, 3696–3713 (2015).

36. J. Wang, R. M. Wolf, J. W. Caldwell, P. A. Kollman, D. A. Case, Development and testing of a general amber force field. *Journal of Computational Chemistry* **25**, 1157–1174 (2004).

37. A. Jakalian, D. B. Jack, C. I. Bayly, Fast, efficient generation of high-quality atomic charges. AM1-BCC model: II. *Parameterization and validation. J Comput Chem* **23**, 1623–1641 (2002).

38. A. Klamt, Conductor-like Screening Model for Real Solvents: A New Approach to the Quantitative Calculation of Solvation Phenomena. *J. Phys. Chem.* **99**, 2224–2235 (1995).

39. J. J. Stewart, MOPAC: a semiempirical molecular orbital program. *J Comput Aided Mol Des* **4**, 1–105 (1990).

40. V. Hornak, *et al.*, Comparison of multiple Amber force fields and development of improved protein backbone parameters. *Proteins* **65**, 712–725 (2006).

41. A smooth particle mesh Ewald method - Astrophysics Data System. Available at: https://ui.adsabs.harvard.edu/abs/1995JChPh.103.8577E/abstract [Accessed 9 June 2025].

42. E. Krieger, G. Vriend, New ways to boost molecular dynamics simulations. *Journal of Computational Chemistry* **36**, 996–1007 (2015).

43. J. M. Janz, D. L. Farrens, Role of the retinal hydrogen bond network in rhodopsin Schiff base stability and hydrolysis. *J Biol Chem* **279**, 55886–94 (2004).
